# Upper glycolytic components contribute differently in controlling retinal vascular endothelial cellular behavior: Implications for endothelial-related retinal diseases

**DOI:** 10.1371/journal.pone.0294909

**Published:** 2023-11-30

**Authors:** Nicole Oska, Shaimaa Eltanani, Mohamed Shawky, Armaan Naghdi, Andrew Gregory, Thangal Yumnamcha, Ahmed S. Ibrahim

**Affiliations:** 1 Department of Ophthalmology, Visual, and Anatomical Sciences, School of Medicine, Wayne State University, Detroit, MI, United States of America; 2 Department of Biochemistry, Faculty of Pharmacy, Horus University, New Damietta, Egypt; 3 Department of Biochemistry, Faculty of Pharmacy, Mansoura University, Mansoura, Egypt; 4 Department of Pharmacology, School of Medicine, Wayne State University, Detroit, MI, United States of America; Facultad de Estudios Superiores Iztacala, Universidad Nacional Autónoma de México, MEXICO

## Abstract

**Background:**

Retinal degenerative diseases such as diabetic retinopathy and diabetic macular edema are characterized by impaired retinal endothelial cells (RECs) functionality. While the role of glycolysis in glucose homeostasis is well-established, its contributions to REC barrier assembly and cell spreading remain poorly understood. This study aimed to investigate the importance of upper glycolytic components in regulating the behavior of human RECs (HRECs).

**Methods:**

Electric cell-substrate impedance sensing (ECIS) technology was employed to analyze the real-time impact of various upper glycolytic components on maintaining barrier functionality and cell spreading of HRECs by measuring cell resistance and capacitance, respectively. Specific inhibitors were used: WZB117 to inhibit Glut1/3, lonidamine to inhibit hexokinases, PFK158 to inhibit the PFKFB3-PFK axis, and TDZD-8 to inhibit aldolases. Additionally, the viability of HRECs was evaluated using the lactate dehydrogenase (LDH) cytotoxicity assay.

**Results:**

The most significant reduction in electrical resistance and increase in capacitance of HRECs resulted from the dose-dependent inhibition of PFKFB3/PFK using PFK158, followed by aldolase inhibition using TDZD-8. LDH level analysis at 24- and 48-hours post-treatment with PFK158 (1 μM) or TDZD-8 (1 and 10 μM) showed no significant difference compared to the control, indicating that the disruption of HRECs functionality was not attributed to cell death. Conversely, inhibiting Glut1/3 with WZB117 had minimal impact on HREC behavior, except at higher concentrations (10 μM) and prolonged exposure. Lastly, inhibiting hexokinase with lonidamine did not noticeably alter HREC cell behavior.

**Conclusion:**

This study illustrates the unique impacts of components within upper glycolysis on HREC functionality, emphasizing the crucial role of the PFKFB3/PFK axis in regulating HREC behavior. Understanding the specific contributions of each glycolytic component in preserving normal REC functionality will facilitate the development of targeted interventions for treating endothelial cell dysfunction in retinal disorders while minimizing effects on healthy cells.

## Introduction

Under normal physiological conditions, the retinal endothelium plays a vital role in strengthening the inner blood-retinal barrier (iBRB) to block the entry of fluid and solutes from the circulation into the inner retina [1]. Disruption to the integrity of the iBRB has been linked to the advancement of various retinal vascular diseases [2, 3]. For example, non-proliferative diabetic retinopathy (NPDR), the most common form of DR, is characterized by endothelial cell damage and increased permeability of the iBRB at early stages [4]. As the disease progresses, the damaged blood vessels continue to leak, allowing larger cystoid spaces to develop in the macula (the specialized area in the retina responsible for central vision), resulting in diabetic macular edema (DME) and eventually vision loss [5]. Therefore, a significant amount of research has been conducted to determine the operative mechanisms that maintain the barrier functionality of endothelial cells, and in recent years glucose hemostasis has received considerable attention [6].

**Fig 1:**
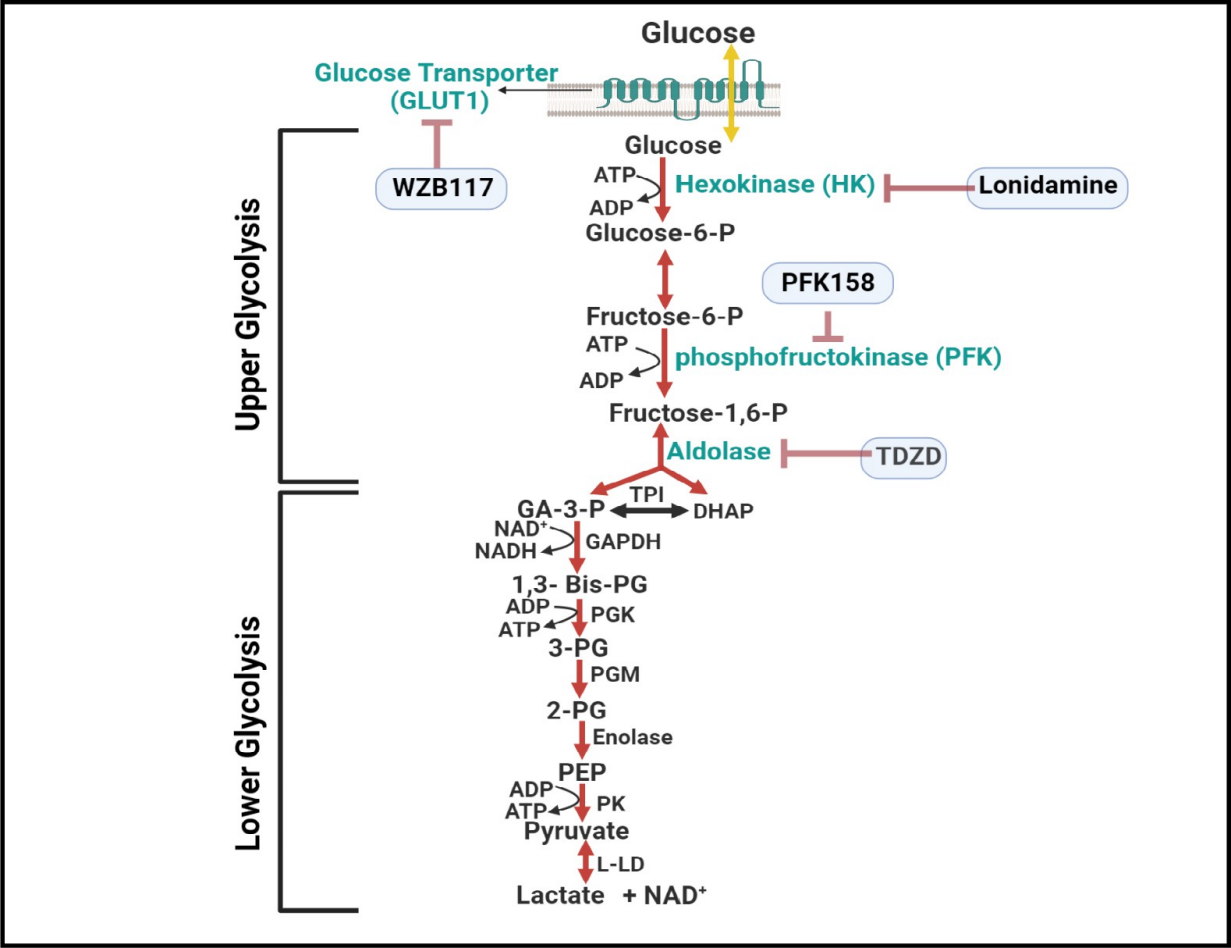
Illustration depicting the glycolysis process in HRECs, distinguishing between upper and lower glycolysis. Upper glycolysis involves the utilization of 2 ATP and begins with the transportation of glucose into HRECs through glucose transporters (Glut). The inhibitors targeting upper glycolytic components, indicated in blue, are WZB117, Lonidamine, PFK158, and TDZD. Abbreviations: GA3P (glyceraldehyde-3-phosphate), TPI (triose phosphate isomerase), DHAP (dihydroacetone phosphate), GAPDH (glyceraldehyde-3-phosphate dehydrogenase), 1,3-Bis-PG (1,3-bisphosphoglycerate), PGK (phosphoglycerate kinase), 3-PG (3-phosphoglycerate), PGM (phosphoglycerate mutase), 2-PG (2-phosphoglycerate), PEP (phosphoenolpyruvate), PK (pyruvate kinase), L-LD (L-lactic dehydrogenase).

The maintenance of glucose homeostasis is regulated by interplay between glycolysis, Krebs cycle, and oxidative phosphorylation (OxPhos). In cell biology, glycolysis is among the most conserved metabolic pathway in prokaryotic and eukaryotic cells [7]. Unlike the Krebs cycle and OxPhos, glycolysis does not require oxygen or compartmentalization by internal membranes, Nobel laureates Embden, Meyerhof, and Parnas [8] elucidated metabolic steps of glycolysis that converts glucose into pyruvate with net production of two adenosine triphosphate (ATP) molecules, dividing it into two parts; the lower part of glycolysis where ATP is produced, preceded by an initial investment of ATP in the upper glycolysis, where glucose is broken down into two trioses: glyceraldehyde 3-phosphate (GA3P) and dihydroxyacetone phosphate (DHAP) by fructose bisphosphate aldolase **(Fig 1)**. Although the endothelium is highly dependent on glycolysis to produce ATP [9], it is unclear how each glycolytic component contributes to the barrier integrity of human retinal endothelial cells (HRECs) and how disruption of these components would differentially affect the barrier functionality. Specifically, the roles of the components in the upper part of glycolysis are of interest, as disruption of upper glycolysis has been implicated in the shunting of glycolytic metabolites into damaging pathways such as the polyol, hexosamine, PKC, and AGEs pathways. These pathways diverge from upper glycolysis at different points. For instance, the polyol pathway generates sorbitol from glucose when hexokinase is saturated, while the hexosamine pathway is triggered by fructose-6-phosphate. The PKC and AGEs pathways arise from disturbances in GA3P metabolism [10].

Glucose entry in HRECs via its transporters is of considerable interest because it is the first rate-limiting step of glucose metabolism and glycolysis. Glucose transporters (Glut)1 and 3 are the most widely distributed glucose transporters and are reported to be concentrated in the endothelium of barrier tissues. 50% of Glut1 resides in cytosolic stores; however, in response to hypoxia or growth factors, Glut1 rapidly translocates to the plasma membrane, where it mediates glucose entry into the endothelium [11, 12]. The recent solving of the human Glut1 and 3 crystal structure [13] enabled the discovery of the selective Glut1/3 inhibitor WZB117 [14], yet the importance of Glut1 and 3 in sustaining the barrier functionality of HREC under physiological conditions still needs to be addressed.

The highly regulated steps of upper glycolysis are the reactions catalyzed by hexokinase and phosphofructokinase (PFK). Hexokinase is the first enzyme in upper glycolysis that phosphorylates glucose into glucose-6-phosphate, an essential step in glucose metabolism. The structures of several hexokinase isoforms have been determined using X-ray crystallography, which enabled the discovery of Lonidamine [15, 16], which has been used to understand the role of hexokinases in different cellular behaviors [17]. However, the relative contribution of hexokinases in maintaining the barrier integrity of HRECs remains largely unknown.

PFK is the second rate-limiting enzyme in upper glycolysis, and its three-dimensional structure was elucidated by X-ray crystallography [18]. The catalytic domain of PFK contains the active site where the phosphorylation of fructose-6-phosphate (F6P) occurs to produce fructose-1,6-bisphosphate (F1,6BP), while the regulatory domain has binding sites for allosteric inhibitory effectors (ATP and citrate) as well as for allosteric activators (fructose-2,6-bisphosphate [F2,6BP] and ADP) [18, 19]. PFKFB (6-phosphofructo-2-kinase/fructose-2, 6-bisphosphatase) is an important enzyme that controls the activity of PFK by regulating the level of F2,6BP, an allosteric activator of PFK. In endothelial cells, F2,6BP is synthesized by PFKFB isoform 3 (PFKFB3) [20], where PFKFB3 expression is tightly regulated by hypoxia-inducible factor (HIF)-1 [21] and vascular endothelial growth factor (VEGF) [22]. PFK158 is a potent and selective inhibitor of PFKFB3 that interferes with PFKFB3 kinase activity to prevent the production of F2,6BP [23]. Although PFKFB3 has been shown to play a major role in the formation of filopodia and lamellipodia in endothelial cells in response to angiogenic stimuli [22], it remains unclear whether PFKFB3 is important for maintaining the barrier integrity of HRECs.

Another critical upper glycolytic component is Aldolase, as it controls the flux of metabolites between the upper and lower parts of glycolysis. Specifically, Aldolase catalyzes the reversible cleavage of F1,6BP into two three-carbon molecules; GA3P and DHAP. The resulting GA3P proceeds through the lower glycolysis pathway to generate pyruvate and produce ATP [8, 24]. TDZD-8 (1,2,4-thiadiazolidine-3,5-dione)-8 has been studied for its potential inhibitory effects on Aldolase enzymes as TDZD-8 covalently modifies the enzyme’s active cysteine residue number-239, which eventually affects several cellular processes that rely on glycolysis [25]. However, there has been little research on the role of Aldolase in maintaining the barrier function of HRECs.

To explore the dynamic responses of barrier-forming cells, such as retinal endothelial cells, to metabolic stressors, the ECIS (electric cell-substrate impedance sensing) system serves as an invaluable tool. Our previous research utilizing ECIS provided compelling evidence that, through uncoupling the electron transport chain from OxPhos and inhibiting Complex I and V, specific components of mitochondrial OxPhos exhibit distinct functions in controlling the cellular behavior of HRECs beyond their role in ATP production [26]. In this current study, our objective is to investigate, in real-time, the influence of upper glycolytic components on regulating HRECs’ cellular behavior, including barrier functionality and spreading over the substrate surface, utilizing the ECIS technology.

## Materials and methods

### ECIS experiment

The effects of two different concentrations (1 μM, and 10 μM) of four different inhibitors that target upper glycolytic components on retinal endothelial cellular behaviors were studied by monitoring the overall cellular impedance (Z) using ECIS®Zθ technology (Applied Biophysics Inc., Troy, NY, USA) as previously described [26, 27]. The four inhibitors were WZB117 (a Glut1/3 inhibitor), lonidamine (a hexokinase inhibitor), PFK158 (a PFKFB3 inhibitor), and TDZD-8 (an aldolase inhibitor). Initially, a 96-well array (96W20idf PET; Applied Biophysics Inc.) was treated with 100 μM cysteine (50 μL/well; Applied Biophysics) for 30 minutes and then aspirated. Afterwards the array was covered with 0.02% gelatin (50 μL/well; Sigma, Burlington, MA, USA) for 30 minutes, followed by aspiration. The next was that HRECs from Cell Systems (Kirkland, WA, USA) were seeded in Microvascular Endothelial Cell Growth Medium-2 BulletKit (Lonza, Walkersville, MD, USA; Catalog #: CC-3202 EGM-2 MV). Once the HRECs became confluent and formed a mature monolayer (capacitance < 20 nF), the culture media were replaced by media free of growth factors for 10–12 hrs before applying different concentrations of the four aforementioned inhibitors. Then the Z of the HREC monolayer was assessed over time and frequency by applying a 1 μA alternating current (AC) to the electrode array located at the base of the 96-well plate. Nine frequencies ranging from 250 to 64,000 Hz were employed to measure Z. To obtain the normalized Z value at each time point, the raw Z value obtained at each time point was divided by the Z value obtained prior to treatment with glycolytic inhibitors and plotted against time. Additionally, ECIS software was utilized to calculate the resistance (R) and capacitance (C) across the HREC monolayer based on the measured Z. These data points were either collected at the end or throughout the experiment by calculating the area under the curve (AUC). For transfection, the seeded HRECs underwent transfection using the TriFECTa® RNAi Kit designed for Glut1 (hs.Ri.SCL2A1.13), PFKFB3 (hs.Ri.PFKFB3.13.1,3), or control Dicer-substrate RNA (DsiRNA) duplexes (Integrated DNA Technologies) using lipofectamine 2000 (Invitrogen) per the manufacturer’s instructions.

### Assessment of HREC viability

The effect of different concentrations of the four different inhibitors on cell viability of HRECs was determined by lactate dehydrogenase (LDH) Cytotoxicity Assay (CyQUANT™; Invitrogen-C20300, Waltham, MA, USA). This method employed the following: HRECs were cultured in 96-well plates (1 × 10^4^/well), and after cells became confluent, the culture media were replaced by media free of growth factors for 10–12 h before applying different concentrations of the four inhibitors (0, 1 and 10 μM) for 24 and 48 hrs. Subsequently, the amount of LDH released into the medium was determined by following the manufacturer’s instructions.

### Western blot analysis

Briefly, HRECs were resuspended in RIPA lysis buffer containing protease and phosphatase inhibitors as previously described [28]. Glut1 protein expression was detected by anti-Glut1 antibody (Abcam, Catalog # ab14683) per the manufacture’s instruction of not boiling the cell lysate to avoid protein aggregations of Glut1. PFKFB3 and β-actin protein expressions were detected by anti- PFKFB3 and β-actin antibodies, respectively (Cell signaling, Catalog # 13123 and Santa Cruz, Catalog # sc-47778, respectively) after boiling the samples. All the primary antibodies incubations were followed by a horseradish peroxidase-conjugated secondary antibody and enhanced chemiluminescence detection system (Thermo Fisher Scientific, Rockford, IL, USA). iBright Imaging Systems (Thermo Fisher Scientific) was used to take blot images, and protein expression was quantified by Image J software.

### Statistical analysis

Statistical comparisons among the experimental groups were performed using one-way analysis of variance (ANOVA). To address multiple comparisons, the False Discovery Rate (FDR) was controlled using the two-stage linear step-up procedure of Benjamin, Krieger, and Yekitieli, with a threshold of <0.05. Statistical comparisons among siRNA transfection experimental groups were performed using unpaired t-test analyses. The p-values were visually depicted as follows: * for p ≤ 0.05, ** for p ≤ 0.01, *** for p ≤ 0.001, and **** for p ≤ 0.0001. The figure legends included the number of biological replicates for each analysis.

## Results

### Real-time monitoring the effect of different upper glycolytic components on HREC bioimpedance

**Fig 2:**
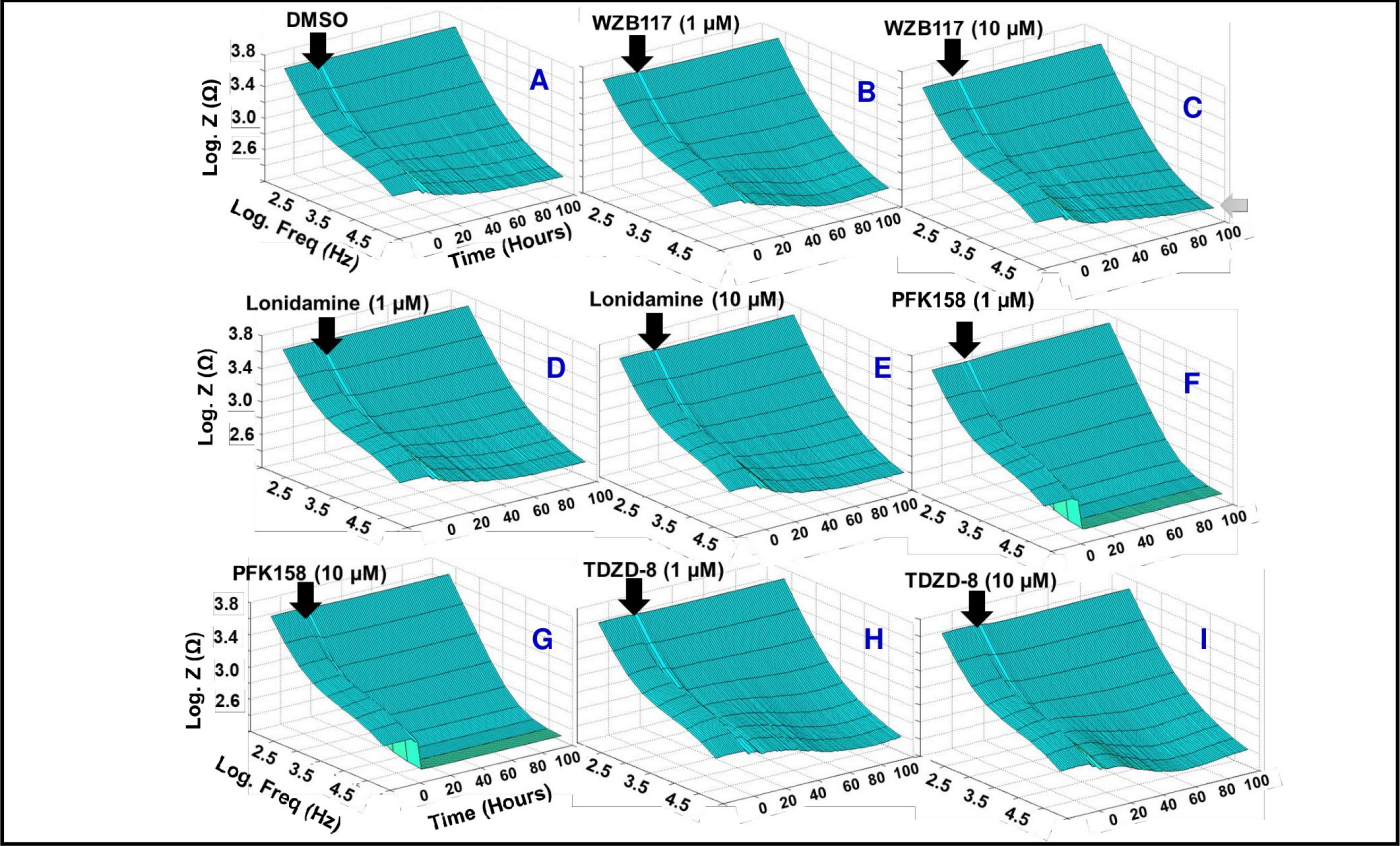
Bioimpedance analysis of the impact of various upper glycolytic inhibitors on the barrier function of HRECs using ECIS technology. Three-dimensional plots illustrating the logarithm of impedance (Z) measured in ohms (Ω) over time and the logarithm of the 1 μA alternating current (AC). **(A)** HRECs treated with the vehicle DMSO. **(B and C)** HRECs treated with 1 μM and 10 μM Glut1/3 inhibitor (WZB117), respectively. **(D and E)** HRECs treated with 1 μM and 10 μM HK inhibitor (Lonidamine), respectively. **(F and G)** HRECs treated with 1 μM and 10 μM PFK (PFKFB3 inhibitor), respectively. **(H and I)** HRECs treated with 1 μM and 10 μM TDZD-8 (aldolase inhibitor), respectively. The treatment of HRECs commenced at t = 0 hours when a confluent monolayer was formed. The impedance value (Z0) at t = 0 hours was used to normalize all other impedance measurements (Zt) using the ratio Zt/Z0. Real-time impedance measurement was conducted using different AC frequencies ranging from 250 to 64,000 Hz. Abbreviations: Z (impedance), Norm (normalized), Freq (frequency), Zt (impedance at time t), Z0 (impedance at time 0).

To investigate the importance of different glycolytic components in maintaining the barrier function of HRECs, we performed bioimpedance analysis using the ECIS technology. Specifically, we used inhibitors such as WZB117 **(Fig 2B and 2C)** to inhibit Glut1/3, lonidamine **(Fig 2D and 2E)** to inhibit hexokinases, PFK158 **(Fig 2F and 2G)** to inhibit the PFKFB3-PFK axis, and TDZD-8 **(Fig 2H and 2I)** to inhibit aldolases. Various concentrations of these glycolytic inhibitors were added to the HRECs once they reached a plateau in the impedance (Z) curve, indicating the formation of a confluent monolayer (black arrows in **Fig 2**). We continuously monitored the barrier integrity **(Fig 2A–2I)** over a 120-hour period (represented on the z-axis in the 3D model) and across a frequency range from 250 to 64,000 Hz (represented on the x-axis in the 3D model) to determine the effects of treatments with different glycolytic inhibitors on HREC barrier integrity. Among the treatments, PFK158 treatments showed the most visually dramatic decrease in Z post-treatment **(Fig 2F and 2G)**. The WZB117 treatment at 10 μM displayed a noticeable loss in Z towards the end of the experiment (gray arrow in **Fig 2C**), while the TDZD-8 treatment at 10 μM consistently reduced Z throughout the experiment, which recovered towards the end **(Fig 2I)**. In contrast, a lower concentration (1 μM) of WZB117 **(Fig 2B)** or TDZD-8 **(Fig 2H)** did not affect the Z of HRECs compared to the control group **(Fig 2A)** treated with the vehicle (DMSO). Interestingly, lonidamine treatments at both 1 μM and 10 μM showed no discernible differences in Z compared to the control treatment **(Fig 2D and 2E)**. These findings highlight the distinct contributions of Glut1/3, Hexokinase, PFK, and aldolase in preserving the barrier functionality of HRECs. We have further explored and investigated these roles in subsequent experiments to gain a deeper understanding.

### The effect of Glut1/3 inhibition on the behavior of HRECs

**Fig 3:**
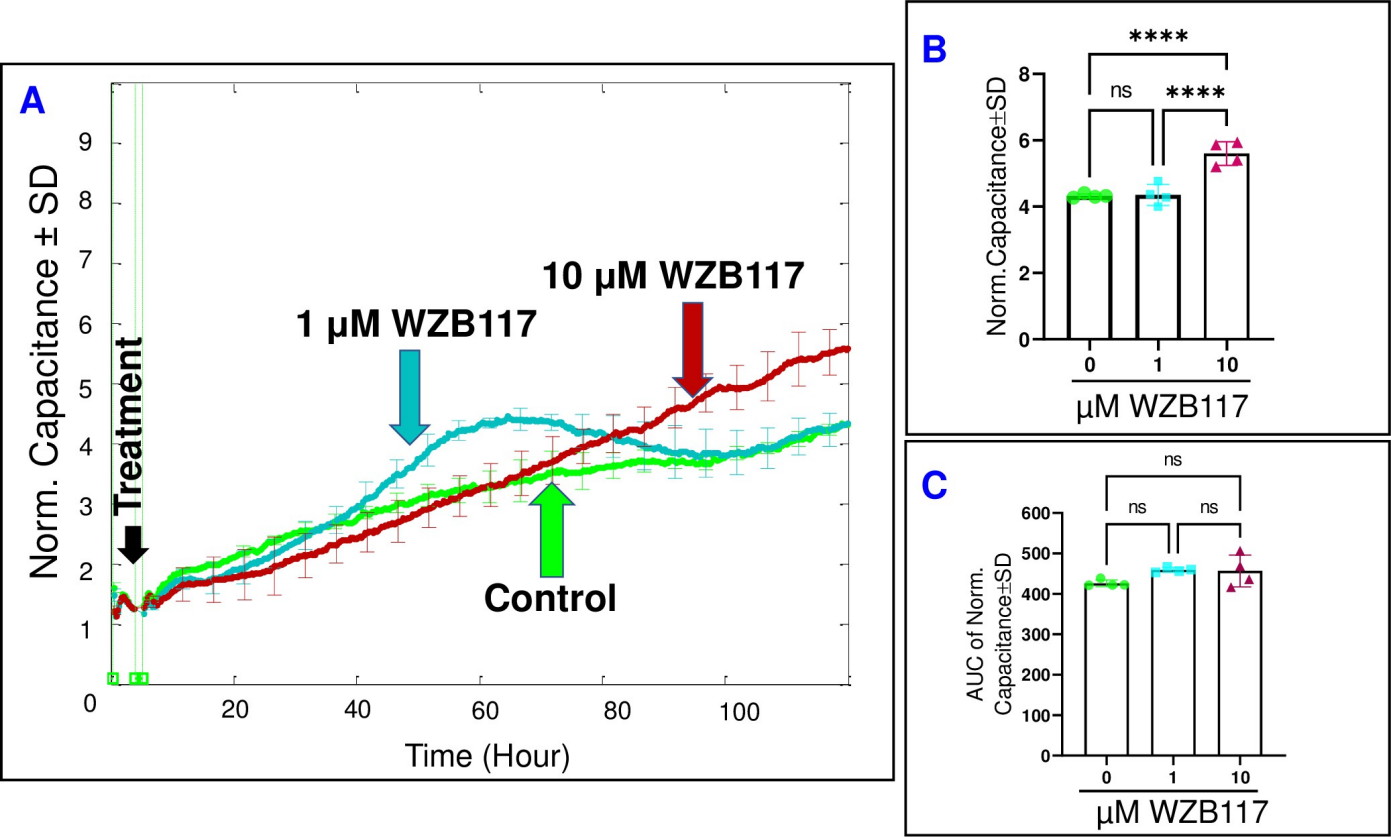
Real-time spread of HRECs over the electrode in response to Glut1/3 inhibitor (WZB117). **(A)** The plot shows the normalized capacitance across HRECs over time, measured at an AC frequency of 64,000 Hz for both the control group and HRECs treated with WZB117 (1 μM & 10 μM). The capacitance values were normalized using the capacitance measurement taken before the addition of the inhibitor, considered as t = 0 hour. **(B)** The bar graph displays the normalized capacitance for the control group and HRECs treated with WZB117 (1 μM & 10 μM) at the end of the experiment (t = 120 hours). **(C)** The bar graph presents the areas under the normalized capacitance curve for the control group and HRECs treated with WZB117 (1 μM & 10 μM) during the time interval of t = 0–120 hours. Significant changes are indicated by p the value of symbol ****<0.0001; n = 4 biological replicates for each group. Abbreviations: Norm (normalized), AUC (area under the curve), ns (no significance).

From a mathematical perspective, the total Z of HRECs consists of two components: capacitance (C) and resistance (R). These components serve as indicators for different cell behaviors. The C reflects the spreading of cells over the substrate, while the R describes the strength of cell-cell junctions, cell-matrix adherence, and changes in cell morphology. Therefore, our aim was to investigate the significance of Glut1/3 modulation on these cell parameters using the Glut1/3 inhibitor (WZB117). Initially, we examined the effect of WZB117 on the spreading movement of HRECs over the substrate by monitoring the C across HRECs **(Fig 3A)**. To do so, we selected the optimum frequency of 64,000 Hz, which corresponds to the maximum spreading extent of HRECs over the electrode based on our previous publication [27]. Since the C decreases in inverse proportion to the spreading extent, it was crucial to assess the impact of inhibiting Glut1/3 on HRECs’ spreading once the cells reached a state of electrical quiescence. We defined electrical quiescence as the point at which the cells achieved stable minimum plateaus in C curves after being placed on the electrode, which was also determined from our previous publication [27]. The results, depicted in **Fig 3A–3C**, revealed that treatment with WZB117 (1 μM) did not affect the C and, thus, the spreading behavior of HRECs either at the end of the experiment **(Fig 3B)** or throughout its entirety **(Fig 3C)**. Interestingly, it took approximately 100–120 hours for HRECs treated with a higher concentration of WZB117 (10 μM) to exhibit a significant increase in C compared to the control **(Fig 3B)**. However, when comparing the AUCs in **Fig 3C**, neither treatment with WZB117 resulted in a significant increase (29.5%) in C throughout the entire experiment when compared to the control.

**Fig 4:**
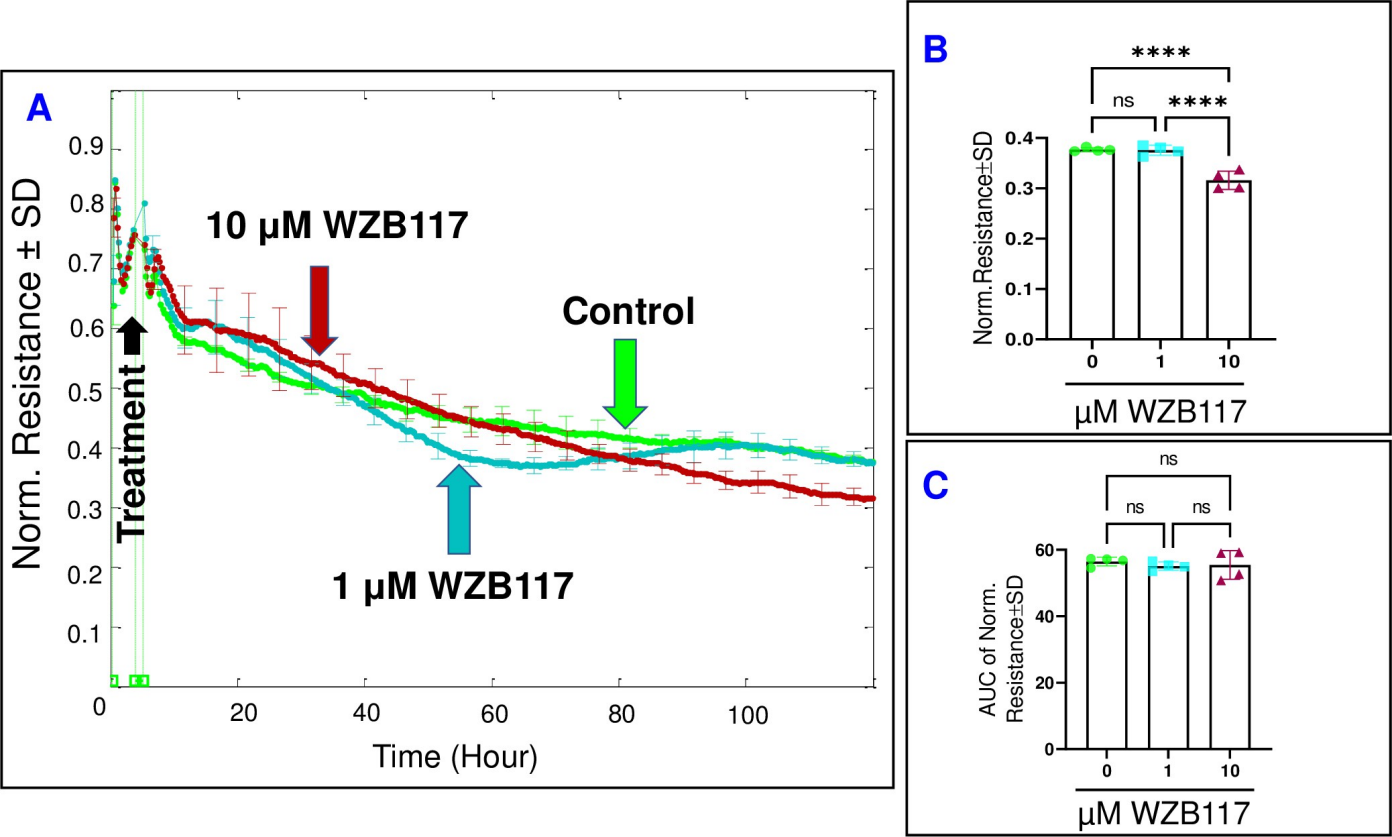
The real-time measurement of the total resistance across HRECs under the effect of the Glut1/3 inhibitor (WZB117). **(A)** The plot illustrates the normalized resistance across HRECs over time, measured at an AC frequency of 4000 Hz for both the control group and HRECs treated with WZB117 (1μM & 10 μM). The resistance was normalized using the measurement taken before the addition of the inhibitor, considered as t = 0 hour. **(B)** The bar graph represents the normalized resistance for the control group and HRECs treated with WZB117 (1μM & 10 μM) at the endpoint of the experiment (t = 120 hours). **(C)** The bar graph displays the areas under the normalized resistance curve for the control group and HRECs treated with WZB117 (1μM & 10 μM) during the time interval of t = 0–120 hours. Significant changes are indicated by the p value of symbol ****<0.0001; n = 4 biological replicates for each group. Abbreviations: Norm (normalized), AUC (area under the curve), ns (no significance).

Next, to determine whether inhibiting Glut1/3 impacts the barrier function of HRECs, the R parameter of the HREC monolayer impedance was measured at a frequency of 4000 Hz, which corresponds to the peak resistance determined from our previous publication [27]. As shown in **Fig 4A**, treatment with WZB117 (1 μM) did not influence the R or the barrier integrity of HRECs at the end of the experiment **(Fig 4B)** or throughout its duration **(Fig 4C)**. However, treatment with a higher concentration of WZB117 (10 μM) led to a decline in R (16.2%) only after prolonged exposure, specifically towards the end of the experiment **(Fig 4B)**, while not affecting R throughout the entire experiment, as indicated by the AUC in **Fig 4C**. These findings suggest that inhibition of Glut1/3 primarily affects HREC behavior only at higher concentrations and following prolonged exposure. These results were confirmed by subsequent transfection of Glut1 siRNA, which revealed that siRNA silencing of Glut1 significantly decreased R of HRECs at the end of the experiment (**S1 Fig**).

### The impact of hexokinase inhibition on HREC behavior

**Fig 5:**
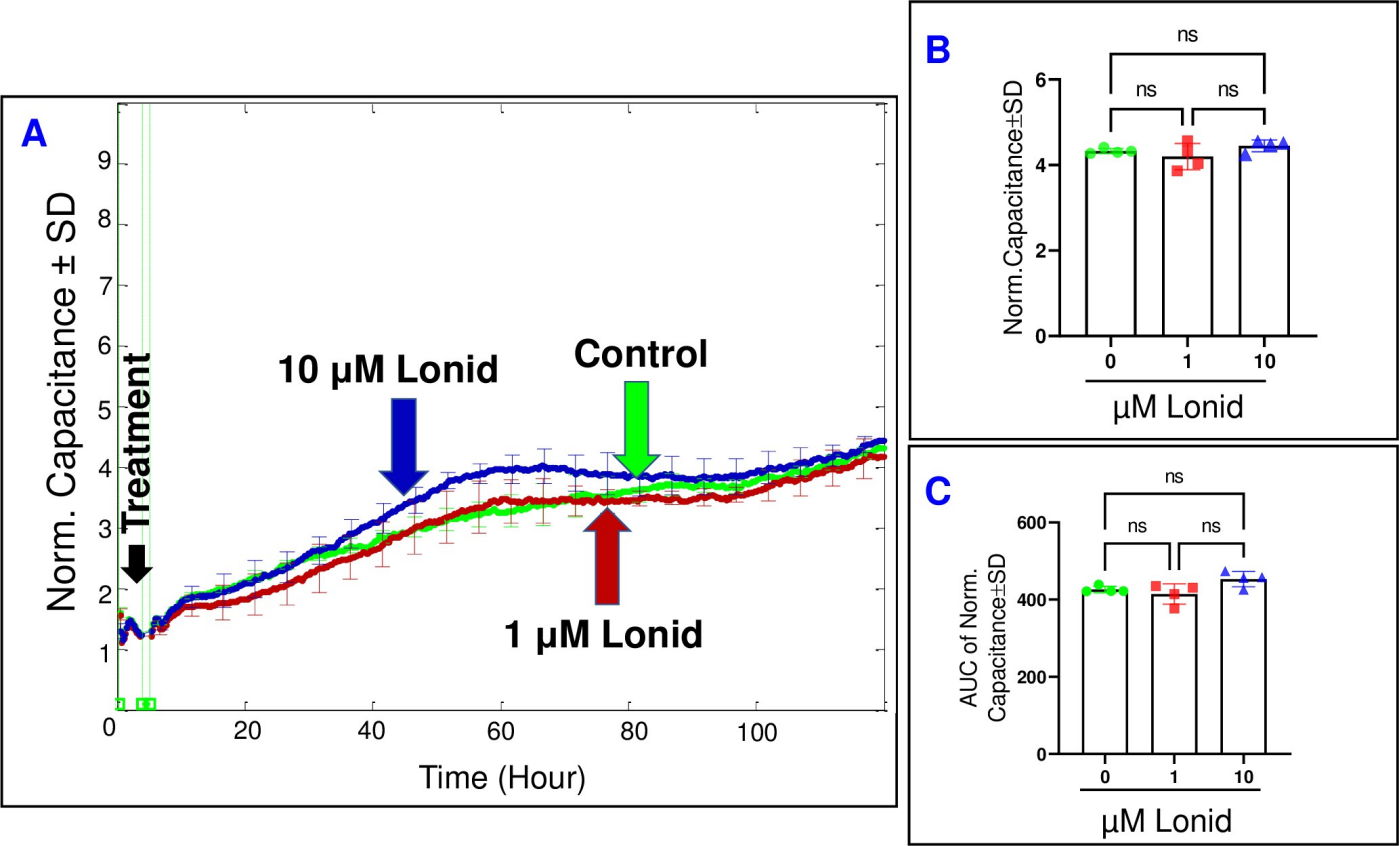
Real-time spread of HRECs over the electrode in response to hexokinase inhibitor (Lonid; lonidamine). **(A)** The plot shows the normalized capacitance across HRECs over time, measured at an AC frequency of 64,000 Hz for both the control group and HRECs treated with Lonid (1 μM & 10 μM). The capacitance values were normalized using the capacitance measurement taken before the addition of the inhibitor, considered as t = 0 hour. **(B)** The bar graph displays the normalized capacitance for the control group and HRECs treated with Lonid (1 μM & 10 μM) at the end of the experiment (t = 120 hours). **(C)** The bar graph presents the areas under the normalized capacitance curve for the control group and HRECs treated with Lonid (1 μM & 10 μM) during the time interval of t = 0–120 hours. n = 4 biological replicates for each group. Abbreviations: Norm (normalized), AUC (area under the curve), ns (no significance).

**Fig 6:**
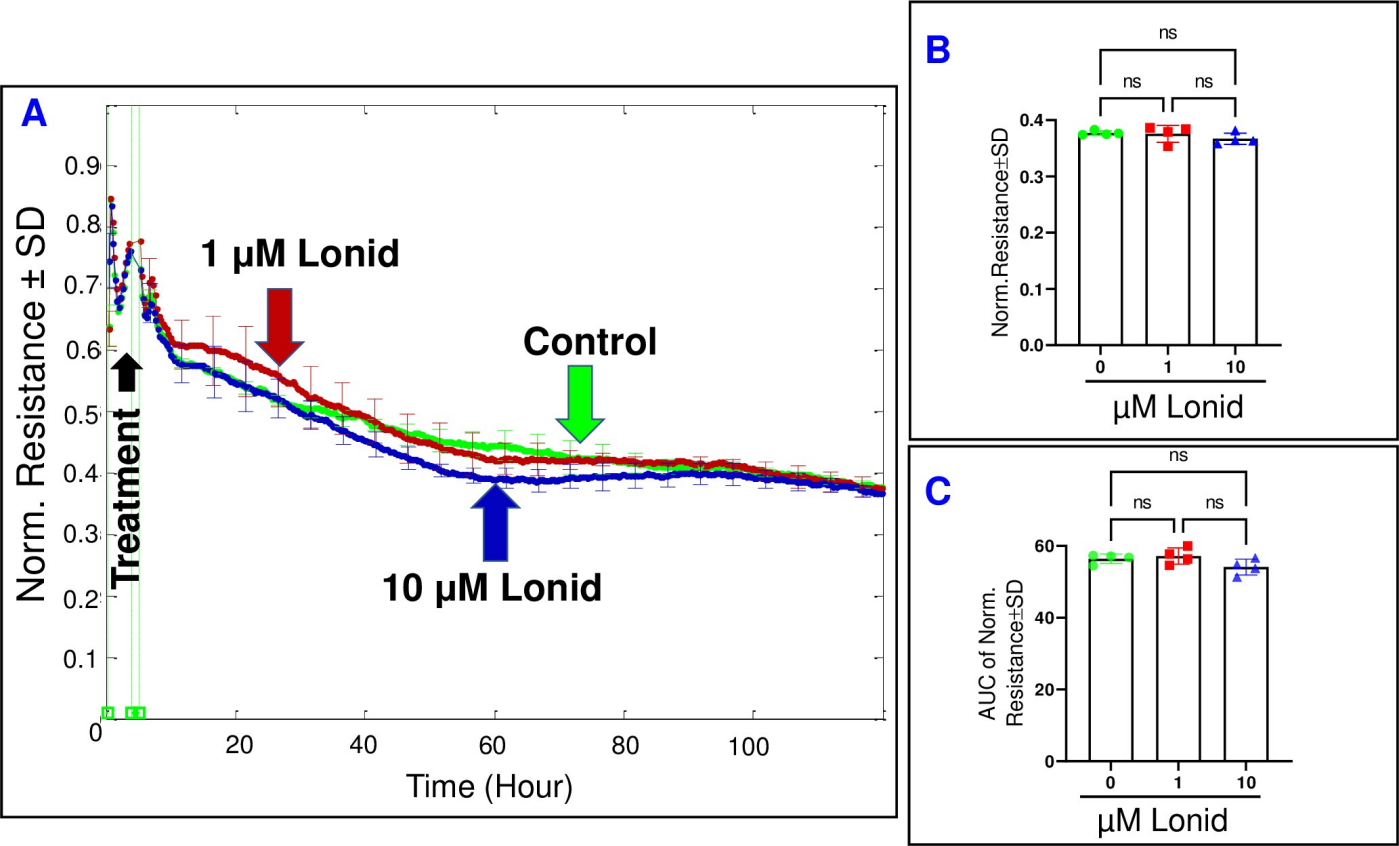
The real-time measurement of the total resistance across HRECs under the effect of the Hexokinase inhibitor (Lonid; lonidamine). **(A)** The plot illustrates the normalized resistance across HRECs over time, measured at an AC frequency of 4000 Hz for both the control group and HRECs treated with Lonid (1μM & 10 μM). The resistance was normalized using the measurement taken before the addition of the inhibitor, considered as t = 0 hour. **(B)** The bar graph represents the normalized resistance for the control group and HRECs treated with Lonid (1μM & 10 μM) at the endpoint of the experiment (t = 120 hours). **(C)** The bar graph displays the areas under the normalized resistance curve for the control group and HRECs treated with Lonid (1μM & 10 μM) during the time interval of t = 0–120 hours. n = 4 biological replicates for each group. Abbreviations: Norm (normalized), AUC (area under the curve), ns (no significance).

We subsequently assessed the influence of inhibiting Hexokinase on the behavior of HRECs using the Hexokinase inhibitor, lonidamine. Firstly, we examined the effect of lonidamine on the spreading behavior of HRECs by monitoring the response of C across HRECs at a frequency of 64,000 Hz over time (**Fig 5A**). The C of HRECs did not show any significant changes following treatment with either lonidamine concentration (1 or 10 μM) at the end of the 120-hour experiment (**Fig 5B**). Furthermore, the AUC for each C response was calculated to evaluate the effect of lonidamine on the C throughout the duration of the experiment. As shown in **Fig 5C**, the AUCs for both lonidamine groups remained insignificantly different from the control. Secondly, we examined the impact of lonidamine on the barrier function of HRECs by monitoring the R response across the cells at a frequency of 4000 Hz over time. As depicted in **Fig 6A**, no notable reductions were observed in the R curves at the end of the experiment (**Fig 6B**) or throughout the experimental duration (**Fig 6C**) following treatment with 1 or 10 μM of lonidamine. These findings provide evidence that Hexokinase does not play a significant role in regulating the behavior of HRECs.

### The impact of PFKFB3/PFK inhibition on HREC behavior

**Fig 7:**
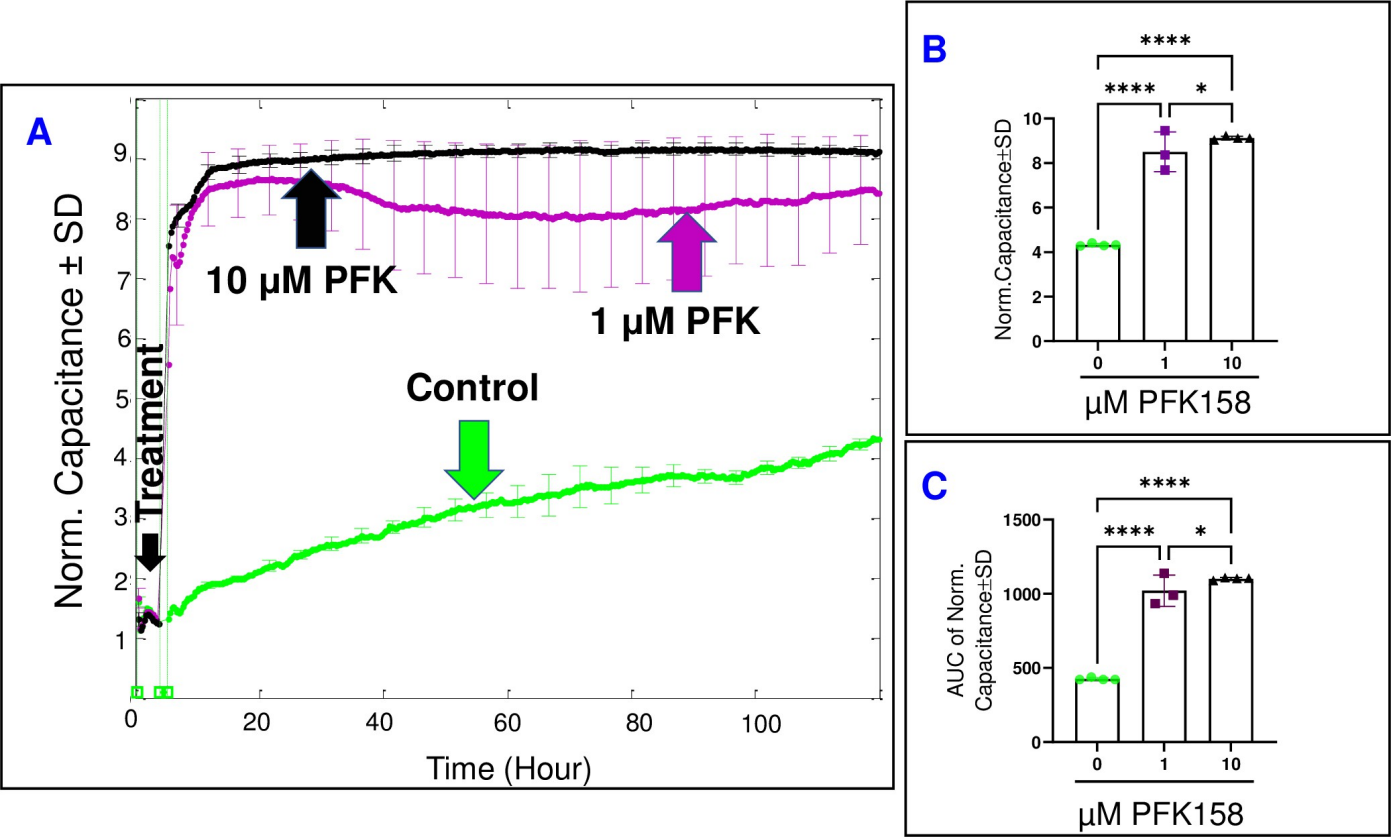
Real-time spread of HRECs over the electrode in response to the PFKFB3-PFK inhibitor (PFK158). **(A)** The plot shows the normalized capacitance across HRECs over time, measured at an AC frequency of 64,000 Hz for both the control group and HRECs treated with PFK158 (1 μM & 10 μM). The capacitance values were normalized using the capacitance measurement taken before the addition of the inhibitor, considered as t = 0 hour. **(B)** The bar graph displays the normalized capacitance for the control group and HRECs treated with PFK158 (1 μM & 10 μM) at the end of the experiment (t = 120 hours). **(C)** The bar graph presents the areas under the normalized capacitance curve for the control group and HRECs treated with PFK158 (1 μM & 10 μM) during the time interval of t = 0–120 hours. Significant changes are indicated by p values of symbol ****<0.0001 and *<0.05; n = 3–4 biological replicates for each group. Abbreviations: Norm (normalized) and AUC (area under the curve).

After having shown that the inhibition of PFKFB3/PFK by PFK158 altered the bioimpedance signature of HRECs largely and rapidly **(Fig 2F and 2G)**, interest has been expanded to dissect the effect of inhibiting PFKFB3/PFK on the parameters that govern the total bioimpedance of HRECs, which include C and R. According to **Fig 7A**, the C of HRECs exhibited a rapid acceleration following treatments with PFK158 (1 and 10 μM), causing a dose-dependent increase in the C (96.5% and 110.9%, respectively) towards the end of the experiment **(Fig 7B)**. To assess whether the effects of PFK158 on the C were dose-dependent throughout the entire experiment, the AUC was calculated for each C curve **(Fig 7C)**. Notably, each PFK158 dose displayed a significant difference in their AUCs compared to each other or to the control group (139.5% and 158.5% increase with 1 μM and 10 μM, respectively), which supports the notion that inhibiting PFKFB3/PFK had a dose-dependent impact on compromising the spreading behavior of HRECs over the substrate not only at the end of the experiment but also throughout the entire experimental duration.

**Fig 8:**
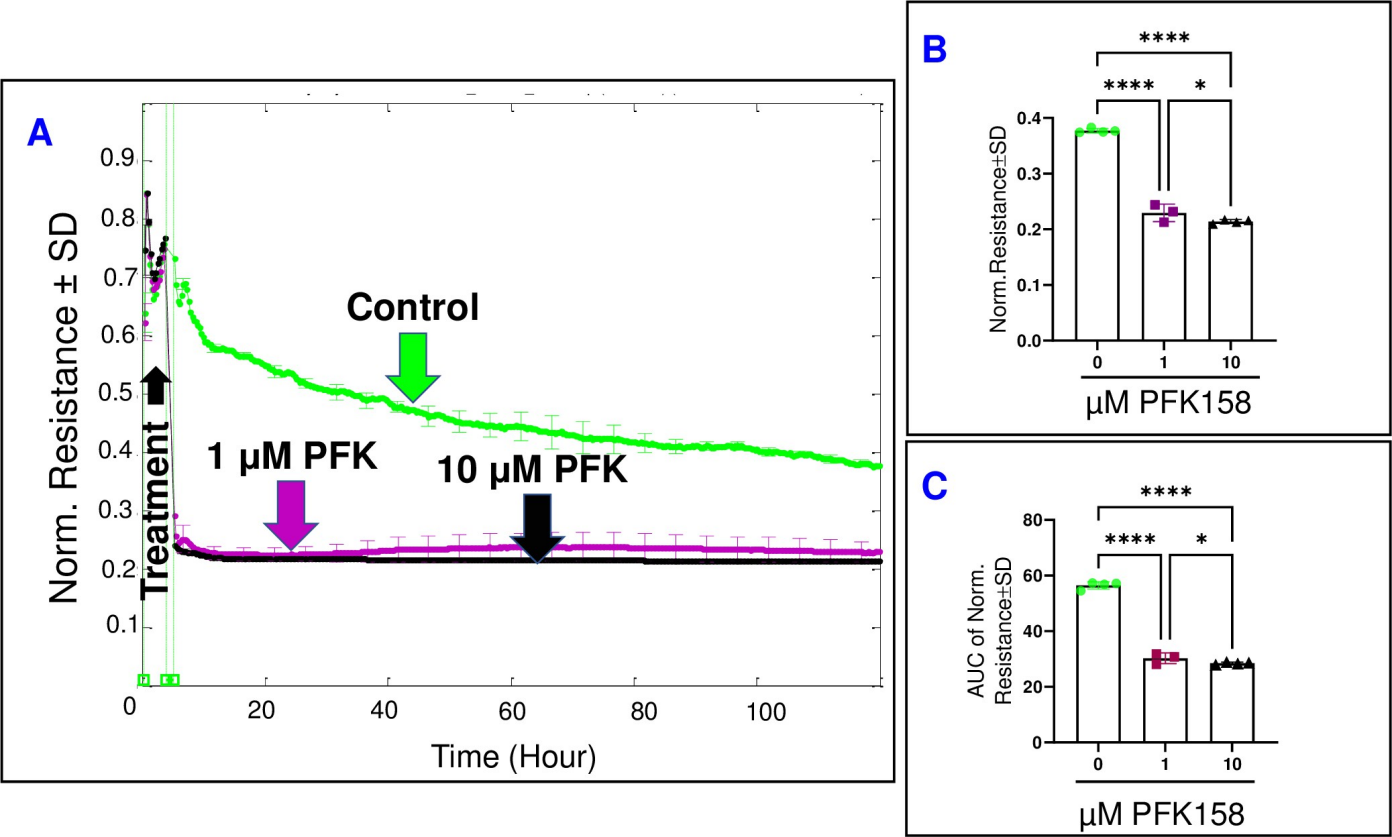
The real-time measurement of the total resistance across HRECs under the effect of the PFKFB3-PFK inhibitor (PFK158). **(A)** The plot illustrates the normalized resistance across HRECs over time, measured at an AC frequency of 4000 Hz for both the control group and HRECs treated with PFK158 (1μM & 10 μM). The resistance was normalized using the measurement taken before the addition of the inhibitor, considered as t = 0 hour. **(B)** The bar graph represents the normalized resistance for the control group and HRECs treated with PFK158 (1μM & 10 μM) at the endpoint of the experiment (t = 120 hours). **(C)** The bar graph displays the areas under the normalized resistance curve for the control group and HRECs treated with PFK158 (1μM & 10 μM) during the time interval of t = 0–120 hours. Significant changes are indicated by p values of symbol ****<0.0001 and *<0.05; n = 3–4 biological replicates for each group. Abbreviations: Norm (normalized) and AUC (area under the curve).

Next, we aimed to determine the impact of PFK158 on the overall barrier functionality of HRECs by evaluating the total R across the HREC monolayer at a frequency of 4000 Hz. The HREC groups were subjected to different concentrations of PFK158, and their total R values vs. time were measured for a duration of 120 hours **(Fig 8)**. Interestingly, the inhibition of PFKFB3/PFK through PFK158 treatment exhibited a dose-dependent decrease in R, observed both at the end of the experiment (39.2% and 43.2% decrease with 1 μM and 10 μM, respectively) **(Fig 8B)** and consistently throughout the entire experimental period (46.4% and 49.6% decrease 1 μM and 10 μM, respectively) **(Fig 8C)**. Therefore, it can be inferred that the inhibition of PFKFB3/PFK has a significantly detrimental and irreversible effect on the barrier function of HRECs. These results were confirmed by subsequent transfection of PFKFB3 siRNA, which revealed that siRNA silencing of PFKFB3 significantly decreased R of HRECs (**S2 Fig**).

### The effect of aldolase inhibition on the behavior of HRECs

**Fig 9:**
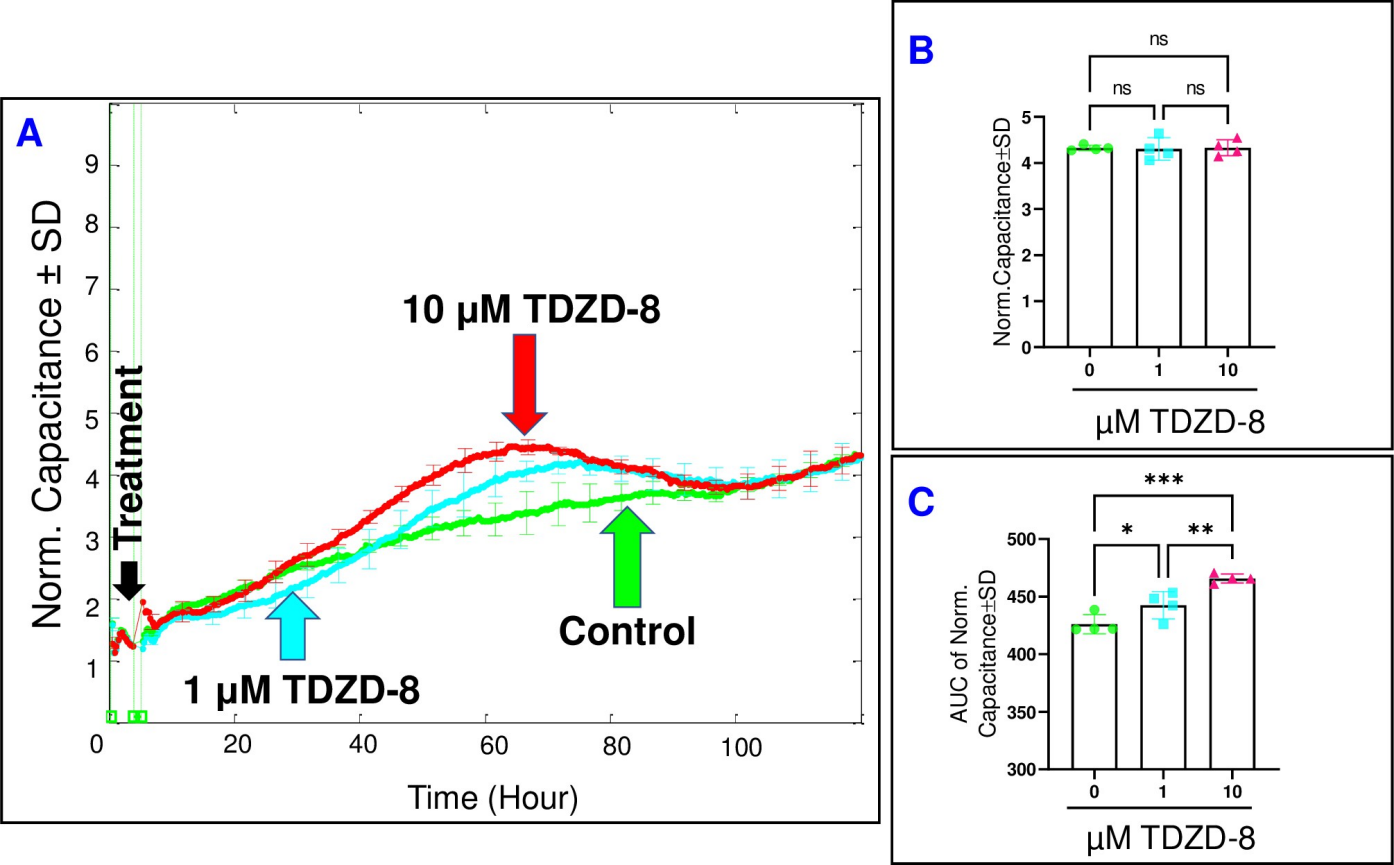
Real-time spread of HRECs over the electrode in response to the aldolase inhibitor (TDZD-8). **(A)** The plot shows the normalized capacitance across HRECs over time, measured at an AC frequency of 64,000 Hz for both the control group and HRECs treated with TDZD-8 (1 μM & 10 μM). The capacitance values were normalized using the capacitance measurement taken before the addition of the inhibitor, considered as t = 0 hour. **(B)** The bar graph displays the normalized capacitance for the control group and HRECs treated with TDZD-8 (1 μM & 10 μM) at the end of the experiment (t = 120 hours). **(C)** The bar graph presents the areas under the normalized capacitance curve for the control group and HRECs treated with TDZD-8 (1 μM & 10 μM) during the time interval of t = 0–120 hours. Significant changes are indicated by p values of symbol ***<0.001, **<0.01, and *<0.05; n = 4 biological replicates for each group. Abbreviations: Norm (normalized) and AUC (area under the curve).

**Fig 10:**
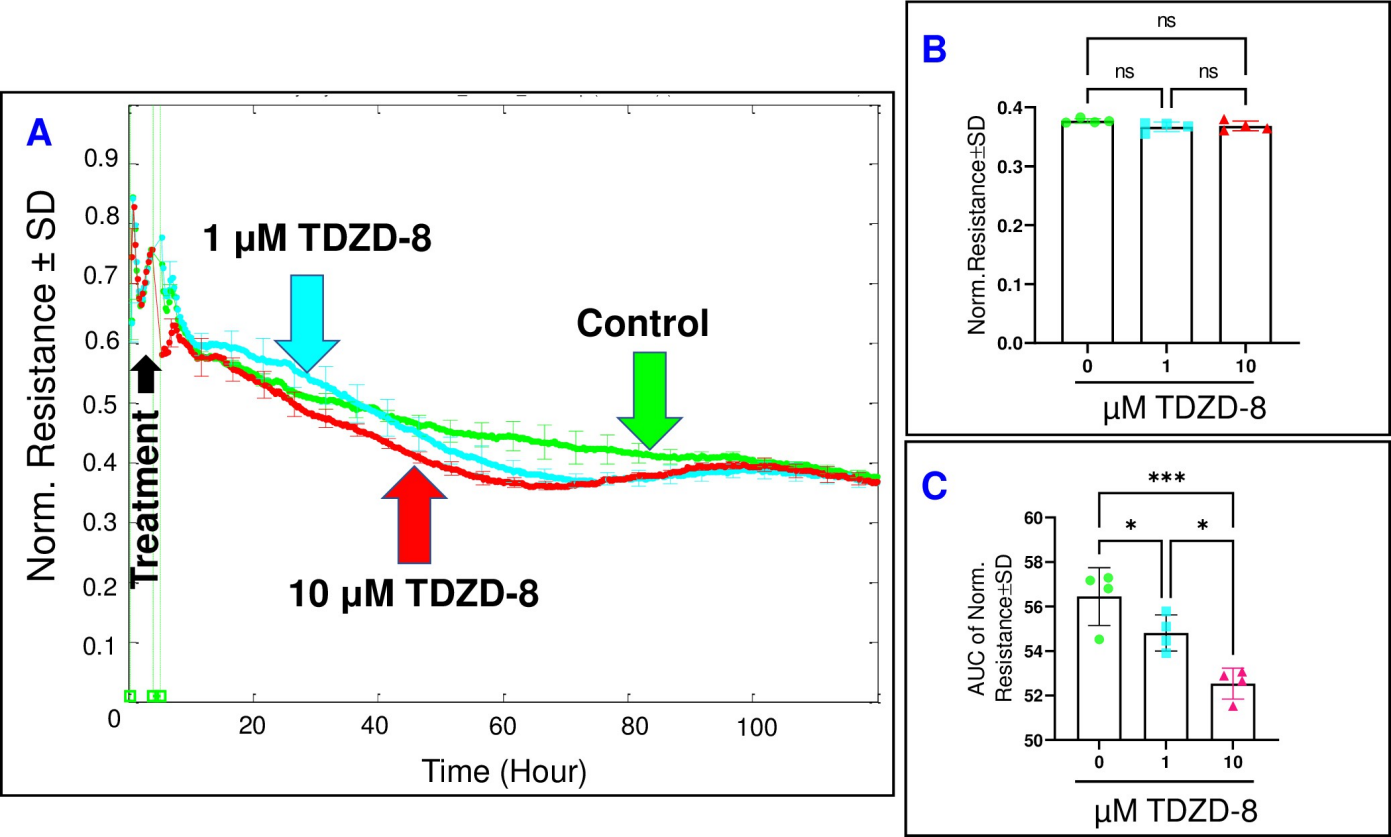
The real-time measurement of the total resistance across HRECs under the effect of the aldolase inhibitor (TDZD-8). **(A)** The plot illustrates the normalized resistance across HRECs over time, measured at an AC frequency of 4000 Hz for both the control group and HRECs treated with TDZD-8 (1μM & 10 μM). The resistance was normalized using the measurement taken before the addition of the inhibitor, considered as t = 0 hour. **(B)** The bar graph represents the normalized resistance for the control group and HRECs treated with TDZD-8 (1μM & 10 μM) at the endpoint of the experiment (t = 120 hours). **(C)** The bar graph displays the areas under the normalized resistance curve for the control group and HRECs treated with TDZD-8 (1μM & 10 μM) during the time interval of t = 0–120 hours. Significant changes are indicated by p values of symbol ****<0.0001 and *<0.05; n = 4 biological replicates for each group. Abbreviations: Norm (normalized) and AUC (area under the curve).

In contrast to the impact of PFKFB3/PFK inhibition, the inhibition of aldolase using TDZD-8 exhibited a significantly lesser influence on the C of HRECs, as observed in **Fig 9A**. Neither of the TDZD-8 treatments affected the final C of HRECs at the end of the experiment, as depicted in **Fig 9B**. However, a comparison of the AUCs in **Fig 9C** reveals a dose-dependent increase in capacitance throughout the entire experiment when TDZD-8 was administered (3.8% and 9.3% increase with 1 μM and 10 μM, respectively). This suggests that the impact of aldolase inhibition on the spreading of HRECs is reversible. Next, we proceeded to evaluate the impact of TDZD-8 on the barrier function of HRECs by monitoring the changes in R of HRECs at 4000 Hz over time **(Fig 10A)**. Interestingly, the administration of TDZD-8 at both 1 μM and 10 μM did not result in significant decreases in the R of HREC when measured at the end of the experiment **(Fig 10B)**. However, both concentrations of TDZD-8 induced a notable and dose-dependent reduction in R compared to the control shortly after treatments (2.9% and 6.9% decrease with 1 μM and 10 μM, respectively). As the experiment progressed, there was a subsequent recovery of R towards the end **(Fig 10A and 10C)**. These findings suggest that while the initial impact of aldolase inhibition affects the integrity of the HREC barrier, it exhibits a recoverable nature in the long run.

### Assessing the viability of HRECs with different upper glycolytic inhibitors

**Fig 11:**
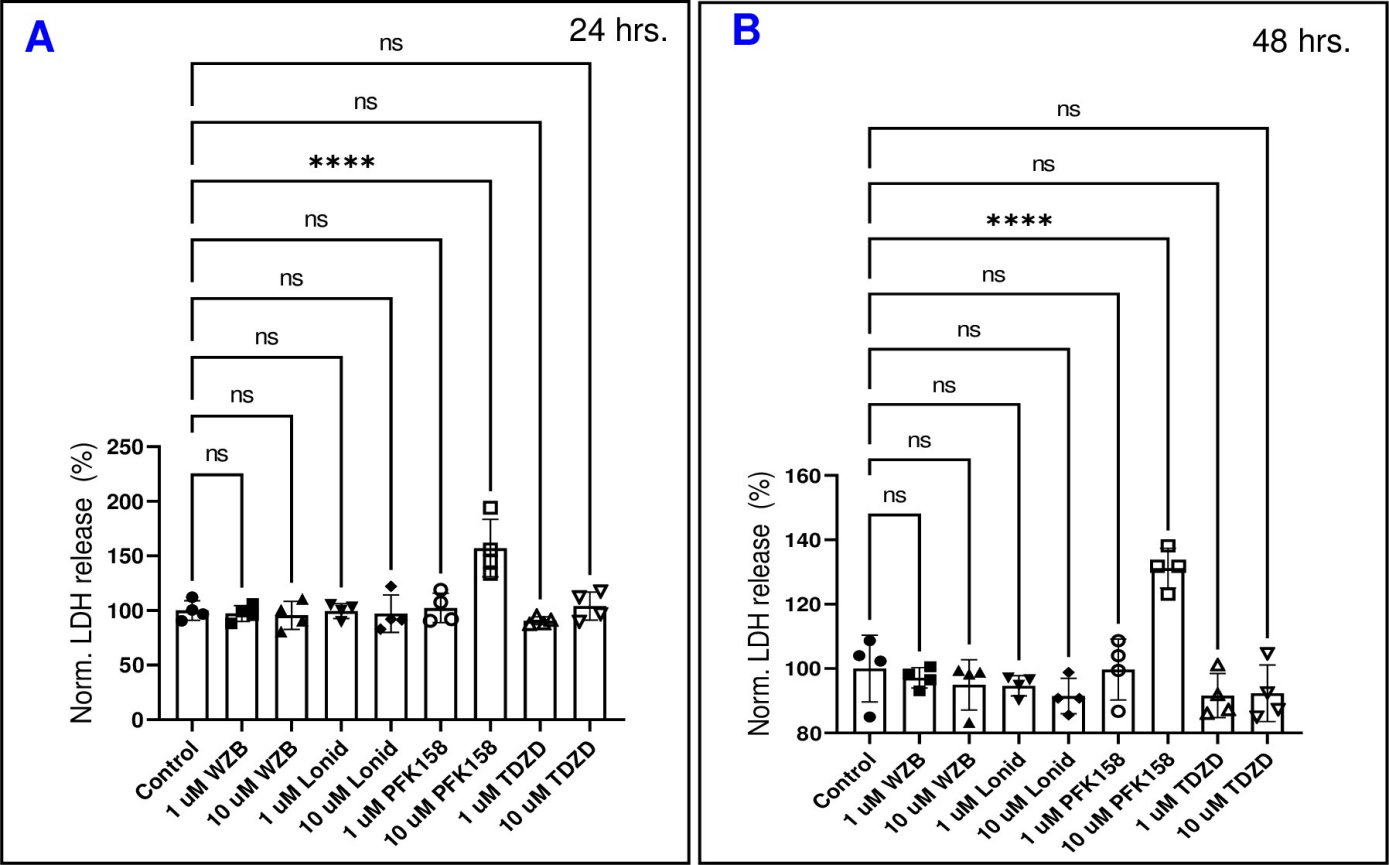
The effects of glycolytic inhibitors on HRECs viability. Lactate dehydrogenase (LDH) assays were conducted at 24 hours and 48 hours for both the control group and cells treated with various upper glycolytic inhibitors. HRECs death was monitored by measuring the amount of LDH released in the culture media. **(A)** The impact of different glycolytic inhibitors on HRECs viability at 24 hours. **(B)** The impact of different glycolytic inhibitors on HRECs viability at 48 hours. Significant changes are indicated by the p value of symbol ****<0.00001; n = 4 biological replicates for each group. Abbreviations: ns (no significance).

To ensure that the observed effects of glycolytic inhibitors on the barrier integrity of HRECs were not due to cell cytotoxicity, we conducted a cytotoxicity assay for all groups of HRECs by measuring lactate dehydrogenase (LDH) release. The time frame selected for analysis was 24–48 hours after treatment. At both the 24-hour and 48-hour marks, only PFK158 (10 μM) caused a significant increase in LDH release, indicating cytotoxicity, while PFK158 (1 μM) and TDZD-8 (1 and 10 μM) did not exhibit any cytotoxic effects **(Fig 11A and 11B)**. This contrasts with the observations in **Figs 8A** and **10A**, where clear reductions in total resistance were already evident in the PFK158 (1 μM) group **(Fig 8A)** and both TDZD-8 groups (**Fig 10A**) at this 24-48-hour time interval. Notably, the resistance of the PFK158 (1 μM) group had reached a minimum level several hours prior to this time interval, yet **Fig 11** demonstrates that this group had not lost viability. Taken together, these findings indicate that the disruption of HRECs’ barrier integrity is an early event that occurs in response to PFKFB3/PFK and aldolase inhibition, preceding any noticeable effects on cell viability.

## Discussion

The key finding of this study is that the components involved in upper glycolysis exhibit distinct effects on HREC functionality, with PFKFB3/PFK playing a crucial role in regulating both the barrier functionality and spreading behavior of HRECs, extending beyond its role in ATP production. The following results support this conclusion: Firstly, inhibiting PFKFB3/PFK with PFK158 (1 μM) resulted in the most significant reduction in the electrical impedance of HRECs within the first 10 hours, followed by aldolase inhibition using TDZD-8 (1 μM). In contrast, inhibiting Glut1/3 with WZB117 (1 μM) or hexokinase with lonidamine (1 μM) did not show a noticeable decrease in electrical impedance. Secondly, and importantly, the breakdown of HREC barrier function caused by PFKFB3/PFK inhibition was not due to cell death. This was evidenced by the absence of elevated levels of released LDH at 24–48 hours post-administration of PFK158 at 1 μM. Additionally, the breakdown of barrier integrity in HRECs treated with PFK158 was unlikely driven solely by the lack of glycolytic ATP production. This is supported by the minimal impact of lonidamine and TDZD-8, which are other glycolytic inhibitors, on HREC impedance, as well as their minimal effects on R and C parameters. To the best of our knowledge, this study is the first to demonstrate such temporal relationships between HREC barrier parameters in response to inhibitors targeting different upper glycolytic components, using the ECIS mathematical modeling system.

Several studies have indicated that the modulation of PFKFB3/PFK activity leads to various pathological alterations in endothelial cells besides its role in ATP production. For example, the activation of PFKFB3 has been associated with harmful effects on endothelial progenitor cells (EPCs) observed in patients with poor graft function. This involvement occurs by facilitating the expression of the pro-apoptotic transcription factor FOXO3A and its downstream genes, including p21, p27, and FAS [29]. Moreover, PFKFB3 has shown to promote pathological endothelial cell proliferation and vessel sprouting through filopodia/lamellipodia formation and directional migration [30]. Additionally, PFKFB3 activation has been linked to the impairment of endothelial barrier function within the tumor microenvironment by promoting VE-cadherin endocytosis in endothelial cells and reducing the adhesive properties of pericytes through downregulating N-cadherin expression [31]. Conversely, a recent study has shown that inhibiting PFKFB3 exacerbates cellular senescence by compromising the defensive capability against DNA damage in diabetic endothelial cells [32]. These findings collectively provide insight into the complex role of PFKFB3 in endothelial cell biology and its potential implications for pathological conditions. However, there have been no investigations into the contribution of PFKFB3/PFK in regulating retinal endothelial cell behavior under physiological conditions. Our study is the first to demonstrate the crucial role of PFKFB3/PFK in maintaining barrier integrity and spreading coverage of HRECs under normal conditions.

Interestingly, our observations have revealed that treating HRECs with an aldolase inhibitor (TDZD-8) initially compromised both the barrier integrity and the spreading coverage of the cells but over time, the cells were able to overcome these effects. It has been suggested that blocking the enzymatic activity of aldolase results in cells entering a state of imbalanced glycolysis, where upper glycolysis outpaces lower glycolysis, leading to the accumulation of F1,6BP and depletion of its hydrolyzable product (GA3P) [33]. Because GA3P serves as a substrate for lower glycolysis, the decrease in GA3P tends to slow down the velocity of lower glycolysis and ATP production, thereby affecting ATP-dependent cellular processes [34], including those responsible for maintaining endothelial barrier integrity and spreading coverage. These effects can explain the initial dose-dependent decrease in R and increase in C. However, restoring G3P concentration through alternative pathways, such as the glycerol kinase (GK)-mediated phosphorylation of glycerol [35], can reestablish the balance between upper and lower glycolysis by increasing the velocity of lower glycolysis to become equivalent to that of upper glycolysis [34]. This rebalanced state likely explains the observed restoration of barrier integrity and spreading coverage of HRECs following a prolonged period of aldolase inhibition. Taken together, these findings highlight the dynamic interplay between aldolase inhibition, glycolysis imbalance, and the restoration of normal cell behavior in HRECs.

It is important to note that while hexokinase plays a significant role in regulating glycolysis, inhibiting hexokinase with lonidamine in HRECs did not result in any notable differences in cell behavior suggesting that HRECs may utilize alternative pathways to bypass the inhibition of hexokinase. One potential pathway could involve the hydrolysis of glycogen by glycogen phosphorylase, which generates glucose 1-phosphate (G1P) that continues to fuel glycolysis by the enzyme phosphoglucomutase, which converts G1P to G6P [36]. HRECs are known to possess glycogen stores, which serve as an energy reserve during periods of high energy demand or limited nutrient availability, such as hypoxia or nutrient deprivation [37].

Studies have demonstrated that human microvascular endothelial cells, including HRECs, express glucose transporters from the SLC2A family, which are facilitative hexose transporters [38, 39]. Among the 12 members of the Glut family, microvascular endothelial cells primarily express Glut1 and Glut3, with lower expression levels of Gluts 6 and 10. HRECs do not express glucose transporters from the Na+-glucose–linked transporter (SGLT) family, suggesting that glucose enters HRECs mainly through Glut1 and Glut3 [37]. However, our findings indicate that inhibiting Glut1/3 in HRECs did not significantly affect their behavior, except at higher concentrations and with prolonged exposure, suggesting the presence of parallel routes for glucose entry into HRECs. One such alternative pathway involves glycogen, which can undergo degradation through glycophagy by lysophagosomal glucosidases [40]. Recent studies have highlighted Glut6 as a lysosomal glucose transporter involved in transporting glucose from lysosomes into the cytosol [41]. Notably, microvascular endothelial cells express Glut6, although at lower levels compared to Glut1 and Glut3 [37]. Another mechanism explaining the adaptation of HRECs to Glut1/3 inhibition is that around 20% of glucose taken up by the cells is incorporated into glycogen [37]. This glycogen reserve can slowly release glucose into the cytoplasm under stress conditions, including Glut1/3 inhibition. However, when glycogen stores are depleted due to prolonged inhibition of glucose entry, HRECs are unable to sustain their activities. This could explain the significant increase in capacitance and a loss of barrier resistance observed between 100–120 hours in HRECs treated with a higher concentration of WZB117 (10 μM).

A limitation of our study is the absence of an analysis of how the inhibition of upper glycolytic components influences the flow into lower glycolysis and aerobic glycolysis. GAPDH has been identified as the key regulator of the transition from upper glycolysis to lower glycolysis, whereas pyruvate kinase (pyk) primarily governs lower glycolysis [42]. Future studies should investigate in depth the impact of inhibiting upper glycolysis on the activity of these enzymes and on the flux through lower glycolysis. These studies will improve our understanding of how the interplay between upper and lower glycolysis regulates the behavior of HRECs.

To conclude, this study provides insights into the dynamic role of upper glycolytic components in regulating the functionality of HRECs under normal physiological conditions. Specifically, it emphasizes the importance of PFKFB3/PFK in preserving the integrity of the barrier and promoting the coverage of HRECs. These findings present a promising direction for screening potential agents that can enhance the effectiveness of the PFKFB3/PFK pathway, thereby benefiting the clinical treatment of retinal barrier disorders associated with endothelial dysfunction. In future studies aimed at manipulating glycolytic pathways in retinal endothelial cells in various ischemic retinopathies, it is crucial to consider the relative significance of the glycolytic components discussed in this research. Understanding their roles in maintaining the regular activities of retinal endothelial cells under normal conditions will facilitate the development of targeted interventions to address dysfunctional endothelial cells in retinal disorders, while minimizing any adverse effects on normal endothelial cells.

## Supporting information

S1 Fig Real-time measurement of total resistance across HRECs under the influence of Glut1 siRNA. **(A)** Relative Glut1 expression to actin post-transfection with Glut1 siRNA analyzed by Western blot. **(B)** The plot illustrates the resistance across HREC monolayers over time, measured at an AC frequency of 4000 Hz, for both the control group transfected with scramble siRNA (10 nM) and HRECs transfected with Glut1 siRNA (10 nM). **(C)** The bar graph represents normalized resistance for the control group transfected with scramble siRNA (10 nM) and HRECs transfected with Glut1 siRNA (10 nM) at the experiment’s endpoint. Significant changes are denoted by the p-value symbol **<0.01; n = 5 biological replicates for each group.
(TIF)

S2 Fig Real-time measurement of total resistance across HRECs under the effect of PFKFB3 siRNA. **(A)** Relative PFKFB3 expression to actin post-transfection with PFKFB3 siRNA analyzed by Western blot. **(B)** The plot illustrates the resistance across HREC monolayers over time for both the control group transfected with scramble siRNA (10 nM) and HRECs transfected with PFKFB3 siRNA (10 nM). **(C)** The bar graph represents normalized resistance for the control group transfected with scramble siRNA (10 nM) and HRECs transfected with PFKFB3 siRNA (10 nM) at the experiment’s endpoint. Significant changes are denoted by the p-value symbols *<0.05 and ***<0.001; n = 5 biological replicates for each group.
(TIF)

S1 Raw images (PDF)

## Abbreviations

iBRB: inner blood-retinal barrier
DR: diabetic retinopathy
NPDR: non-proliferative diabetic retinopathy
DME: diabetic macular edema
OxPhos: oxidative phosphorylation
ATP: adenosine triphosphate
GA3P: glyceraldehyde-3-phosphate
DHAP: dihydroxyacetone phosphate
HREC: human retinal endothelial cell
Glut: glucose transporter
PFK: phosphofructokinase
F2,6BP: fructose-2,6-bisphosphate
PFKFB: 6-phosphofructo-2-kinase/fructose-2, 6-bisphosphatase
HIF-1: hypoxia-inducible factor 1
VEGF: vascular endothelial growth factor
TDZD-8: 1,2,4-thiadiazolidine-3,5-dione-8
ECIS: electric cell-substrate impedance sensing
Z: cellular impedance
R: resistance
C: capacitance
AUC: area under the curve
LDH: lactate dehydrogenase

## References

[1] Cunha-Vaz J, Bernardes R, Lobo C. Blood-retinal barrier. Eur J Ophthalmol. 2011;21 Suppl 6:S3–9. Epub 2011/01/01. doi: 10.5301/EJO.2010.6049 . 23264323

[2] Diaz-Coranguez M, Ramos C, Antonetti DA. The inner blood-retinal barrier: Cellular basis and development. Vision Res. 2017;139:123–37. Epub 2017/06/18. doi: 10.1016/j.visres.2017.05.009 ; PubMed Central PMCID: PMC5723228. 28619516 PMC5723228

[3] Bharadwaj AS, Appukuttan B, Wilmarth PA, Pan Y, Stempel AJ, Chipps TJ, et al. Role of the retinal vascular endothelial cell in ocular disease. Prog Retin Eye Res. 2013;32:102–80. Epub 2012/09/18. doi: 10.1016/j.preteyeres.2012.08.004 ; PubMed Central PMCID: PMC3679193. 22982179 PMC3679193

[4] Frey T, Antonetti DA. Alterations to the blood-retinal barrier in diabetes: cytokines and reactive oxygen species. Antioxid Redox Signal. 2011;15(5):1271–84. Epub 2011/02/08. doi: 10.1089/ars.2011.3906 . 21294655

[5] Yang J, Liu Z. Mechanistic Pathogenesis of Endothelial Dysfunction in Diabetic Nephropathy and Retinopathy. Front Endocrinol (Lausanne). 2022;13:816400. Epub 2022/06/14. doi: 10.3389/fendo.2022.816400 ; PubMed Central PMCID: PMC9174994. 35692405 PMC9174994

[6] Clyne AM. Endothelial response to glucose: dysfunction, metabolism, and transport. Biochem Soc Trans. 2021;49(1):313–25. Epub 2021/02/02. doi: 10.1042/BST20200611 ; PubMed Central PMCID: PMC7920920. 33522573 PMC7920920

[7] Peregrin-Alvarez JM, Tsoka S, Ouzounis CA. The phylogenetic extent of metabolic enzymes and pathways. Genome Res. 2003;13(3):422–7. Epub 2003/03/06. doi: 10.1101/gr.246903 ; PubMed Central PMCID: PMC430287. 12618373 PMC430287

[8] Kresge N, Simoni RD, Hill RL. Otto Fritz Meyerhof and the elucidation of the glycolytic pathway. J Biol Chem. 2005;280(4):e3. Epub 2005/01/25. . 15665335

[9] Leung SWS, Shi Y. The glycolytic process in endothelial cells and its implications. Acta Pharmacol Sin. 2022;43(2):251–9. Epub 2021/04/15. doi: 10.1038/s41401-021-00647-y ; PubMed Central PMCID: PMC8791959. 33850277 PMC8791959

[10] Yumnamcha T, Guerra M, Singh LP, Ibrahim AS. Metabolic Dysregulation and Neurovascular Dysfunction in Diabetic Retinopathy. Antioxidants (Basel, Switzerland). 2020 Dec 8;9(12):1244. Epub 2020/12/12. doi: 10.3390/antiox9121244 . 33302369 PMC7762582

[11] Kumagai AK, Vinores SA, Pardridge WM. Pathological upregulation of inner blood-retinal barrier Glut1 glucose transporter expression in diabetes mellitus. Brain Res. 1996;706(2):313–7. Epub 1996/01/15. doi: 10.1016/0006-8993(95)01335-0 . 8822374

[12] Kumagai AK. Glucose transport in brain and retina: implications in the management and complications of diabetes. Diabetes Metab Res Rev. 1999;15(4):261–73. doi: 10.1002/(sici)1520-7560(199907/08)15:4<261::aid-dmrr43>3.0.co;2-z . 10495475

[13] Deng D, Xu C, Sun P, Wu J, Yan C, Hu M, et al. Crystal structure of the human glucose transporter GLUT1. Nature. 2014;510(7503):121–5. Epub 2014/05/23. doi: 10.1038/nature13306 . 24847886

[14] Ojelabi OA, Lloyd KP, Simon AH, De Zutter JK, Carruthers A. WZB117 (2-Fluoro-6-(m-hydroxybenzoyloxy) Phenyl m-Hydroxybenzoate) Inhibits GLUT1-mediated Sugar Transport by Binding Reversibly at the Exofacial Sugar Binding Site. J Biol Chem. 2016;291(52):26762–72. Epub 2016/11/12. doi: 10.1074/jbc.M116.759175 ; PubMed Central PMCID: PMC5207184. 27836974 PMC5207184

[15] Aleshin AE, Zeng C, Bartunik HD, Fromm HJ, Honzatko RB. Regulation of hexokinase I: crystal structure of recombinant human brain hexokinase complexed with glucose and phosphate. J Mol Biol. 1998;282(2):345–57. Epub 1998/09/15. doi: 10.1006/jmbi.1998.2017 . 9735292

[16] Sadeghi RN, Karami-Tehrani F, Salami S. Targeting prostate cancer cell metabolism: impact of hexokinase and CPT-1 enzymes. Tumour Biol. 2015;36(4):2893–905. Epub 2014/12/17. doi: 10.1007/s13277-014-2919-4 . 25501281

[17] Brawer MK. Lonidamine: basic science and rationale for treatment of prostatic proliferative disorders. Rev Urol. 2005;7 Suppl 7(Suppl 7):S21–6. Epub 2006/09/21. ; PubMed Central PMCID: PMC1477623. 16986057 PMC1477623

[18] Webb BA, Forouhar F, Szu FE, Seetharaman J, Tong L, Barber DL. Structures of human phosphofructokinase-1 and atomic basis of cancer-associated mutations. Nature. 2015;523(7558):111–4. Epub 2015/05/20. doi: 10.1038/nature14405 ; PubMed Central PMCID: PMC4510984. 25985179 PMC4510984

[19] Ros S, Schulze A. Balancing glycolytic flux: the role of 6-phosphofructo-2-kinase/fructose 2,6-bisphosphatases in cancer metabolism. Cancer Metab. 2013;1(1):8. Epub 2013/11/28. doi: 10.1186/2049-3002-1-8 ; PubMed Central PMCID: PMC4178209. 24280138 PMC4178209

[20] Bando H, Atsumi T, Nishio T, Niwa H, Mishima S, Shimizu C, et al. Phosphorylation of the 6-phosphofructo-2-kinase/fructose 2,6-bisphosphatase/PFKFB3 family of glycolytic regulators in human cancer. Clin Cancer Res. 2005;11(16):5784–92. Epub 2005/08/24. doi: 10.1158/1078-0432.CCR-05-0149 . 16115917

[21] Kassa B, Kumar R, Mickael C, Sanders L, Vohwinkel C, Lee MH, et al. Endothelial cell PHD2-HIF1alpha-PFKFB3 contributes to right ventricle vascular adaptation in pulmonary hypertension. Am J Physiol Lung Cell Mol Physiol. 2021;321(4):L675–L85. Epub 2021/08/05. doi: 10.1152/ajplung.00351.2020 ; PubMed Central PMCID: PMC8560395. 34346780 PMC8560395

[22] Sawada N, Arany Z. Metabolic Regulation of Angiogenesis in Diabetes and Aging. Physiology (Bethesda). 2017;32(4):290–307. Epub 2017/06/16. doi: 10.1152/physiol.00039.2016 ; PubMed Central PMCID: PMC5545609. 28615313 PMC5545609

[23] National Center for Biotechnology Information. PubChem Patent Summary for EP-2831047-A1, Pfkfb3 inhibitor and methods of use as an anti-cancer therapeutic. https://pubchem.ncbi.nlm.nih.gov/patent/EP-2831047-A1. Accessed May 14, 2023.

[24] Dalby A, Dauter Z, Littlechild JA. Crystal structure of human muscle aldolase complexed with fructose 1,6-bisphosphate: mechanistic implications. Protein Sci. 1999;8(2):291–7. Epub 1999/02/27. doi: 10.1110/ps.8.2.291 ; PubMed Central PMCID: PMC2144250. 10048322 PMC2144250

[25] Grandjean G, de Jong PR, James B, Koh MY, Lemos R, Kingston J, et al. Definition of a Novel Feed-Forward Mechanism for Glycolysis-HIF1alpha Signaling in Hypoxic Tumors Highlights Aldolase A as a Therapeutic Target. Cancer Res. 2016;76(14):4259–69. Epub 2016/06/05. doi: 10.1158/0008-5472.CAN-16-0401 ; PubMed Central PMCID: PMC5082982. 27261507 PMC5082982

[26] Eltanani S, Yumnamcha T, Gregory A, Elshal M, Shawky M, Ibrahim AS. Relative Importance of Different Elements of Mitochondrial Oxidative Phosphorylation in Maintaining the Barrier Integrity of Retinal Endothelial Cells: Implications for Vascular-Associated Retinal Diseases. Cells. 2022;11(24). Epub 2022/12/24. doi: 10.3390/cells11244128 ; PubMed Central PMCID: PMC9776835. 36552890 PMC9776835

[27] El-Tanani S, Yumnamcha T, Singh LP, Ibrahim AS. Differential Effects of Cytopathic Hypoxia on Human Retinal Endothelial Cellular Behavior: Implication for Ischemic Retinopathies. Int J Mol Sci. 2022;23(8). Epub 2022/04/24. doi: 10.3390/ijms23084274 ; PubMed Central PMCID: PMC9027301. 35457092 PMC9027301

[28] Guerra MH, Yumnamcha T, Ebrahim AS, Berger EA, Singh LP, Ibrahim AS. Real-Time Monitoring the Effect of Cytopathic Hypoxia on Retinal Pigment Epithelial Barrier Functionality Using Electric Cell-Substrate Impedance Sensing (ECIS) Biosensor Technology. Int J Mol Sci. 2021;22(9). Epub 2021/05/01. doi: 10.3390/ijms22094568 ; PubMed Central PMCID: PMC8123793. 33925448 PMC8123793

[29] Lyu ZS, Tang SQ, Xing T, Zhou Y, Lv M, Fu HX, et al. The glycolytic enzyme PFKFB3 determines bone marrow endothelial progenitor cell damage after chemotherapy and irradiation. Haematologica. 2022;107(10):2365–80. Epub 2022/04/01. doi: 10.3324/haematol.2021.279756 ; PubMed Central PMCID: PMC9521251. 35354250 PMC9521251

[30] Emini Veseli B, Van Wielendaele P, Delibegovic M, Martinet W, De Meyer GRY. The PFKFB3 Inhibitor AZ67 Inhibits Angiogenesis Independently of Glycolysis Inhibition. Int J Mol Sci. 2021;22(11). Epub 2021/06/03. doi: 10.3390/ijms22115970 ; PubMed Central PMCID: PMC8198190. 34073144 PMC8198190

[31] Cantelmo AR, Conradi LC, Brajic A, Goveia J, Kalucka J, Pircher A, et al. Inhibition of the Glycolytic Activator PFKFB3 in Endothelium Induces Tumor Vessel Normalization, Impairs Metastasis, and Improves Chemotherapy. Cancer Cell. 2016;30(6):968–85. Epub 2016/11/22. doi: 10.1016/j.ccell.2016.10.006 ; PubMed Central PMCID: PMC5675554. 27866851 PMC5675554

[32] Sun D, Chen S, Li S, Wang N, Zhang S, Xu L, et al. Enhancement of glycolysis-dependent DNA repair regulated by FOXO1 knockdown via PFKFB3 attenuates hyperglycemia-induced endothelial oxidative stress injury. Redox Biol. 2023;59:102589. Epub 2022/12/29. doi: 10.1016/j.redox.2022.102589 ; PubMed Central PMCID: PMC9803794. 36577299 PMC9803794

[33] Janulevicius A, van Doorn GS. Selection for rapid uptake of scarce or fluctuating resource explains vulnerability of glycolysis to imbalance. PLoS Comput Biol. 2021;17(1):e1008547. Epub 2021/01/20. doi: 10.1371/journal.pcbi.1008547 ; PubMed Central PMCID: PMC7815144. 33465070 PMC7815144

[34] van Heerden JH, Wortel MT, Bruggeman FJ, Heijnen JJ, Bollen YJ, Planque R, et al. Lost in transition: start-up of glycolysis yields subpopulations of nongrowing cells. Science. 2014;343(6174):1245114. Epub 2014/01/18. doi: 10.1126/science.1245114 . 24436182

[35] Mao C, Ozer Z, Zhou M, Uckun FM. X-Ray structure of glycerol kinase complexed with an ATP analog implies a novel mechanism for the ATP-dependent glycerol phosphorylation by glycerol kinase. Biochem Biophys Res Commun. 1999;259(3):640–4. Epub 1999/06/12. doi: 10.1006/bbrc.1999.0816 . 10364471

[36] Adeva-Andany MM, Gonzalez-Lucan M, Donapetry-Garcia C, Fernandez-Fernandez C, Ameneiros-Rodriguez E. Glycogen metabolism in humans. BBA Clin. 2016;5:85–100. Epub 2016/04/07. doi: 10.1016/j.bbacli.2016.02.001 ; PubMed Central PMCID: PMC4802397. 27051594 PMC4802397

[37] Yazdani S, Bilan PJ, Jaldin-Fincati JR, Pang J, Ceban F, Saran E, et al. Dynamic glucose uptake, storage, and release by human microvascular endothelial cells. Mol Biol Cell. 2022;33(12):ar106. Epub 2022/08/04. doi: 10.1091/mbc.E22-04-0146 ; PubMed Central PMCID: PMC9635305. 35921166 PMC9635305

[38] Gaudreault N, Scriven DR, Laher I, Moore ED. Subcellular characterization of glucose uptake in coronary endothelial cells. Microvasc Res. 2008;75(1):73–82. Epub 2007/05/29. doi: 10.1016/j.mvr.2007.04.006 . 17531273

[39] Takagi H, King GL, Aiello LP. Hypoxia upregulates glucose transport activity through an adenosine-mediated increase of GLUT1 expression in retinal capillary endothelial cells. Diabetes. 1998;47(9):1480–8. Epub 1998/09/03. doi: 10.2337/diabetes.47.9.1480 . 9726238

[40] Mandl J, Banhegyi G. The ER ‐ Glycogen Particle ‐ Phagophore Triangle: A Hub Connecting Glycogenolysis and Glycophagy? Pathol Oncol Res. 2018;24(4):821–6. Epub 2018/07/08. doi: 10.1007/s12253-018-0446-0 . 29981013

[41] Lizak B, Szarka A, Kim Y, Choi KS, Nemeth CE, Marcolongo P, et al. Glucose Transport and Transporters in the Endomembranes. Int J Mol Sci. 2019;20(23). Epub 2019/11/28. doi: 10.3390/ijms20235898 ; PubMed Central PMCID: PMC6929180. 31771288 PMC6929180

[42] Shestov AA, Liu X, Ser Z, Cluntun AA, Hung YP, Huang L, et al. Quantitative determinants of aerobic glycolysis identify flux through the enzyme GAPDH as a limiting step. Elife. 2014;3. Epub 2014/07/11. doi: 10.7554/eLife.03342 ; PubMed Central PMCID: PMC4118620. 25009227 PMC4118620

