# Supplementary figures and images for: Upper glycolytic components contribute differently in controlling retinal vascular endothelial cellular behavior: Implications for endothelial-related retinal diseases
Source: PLoS One. 2023 Nov 30;18(11):e0294909. doi: 10.1371/journal.pone.0294909 (PMC10688887; doi:10.1371/journal.pone.0294909)

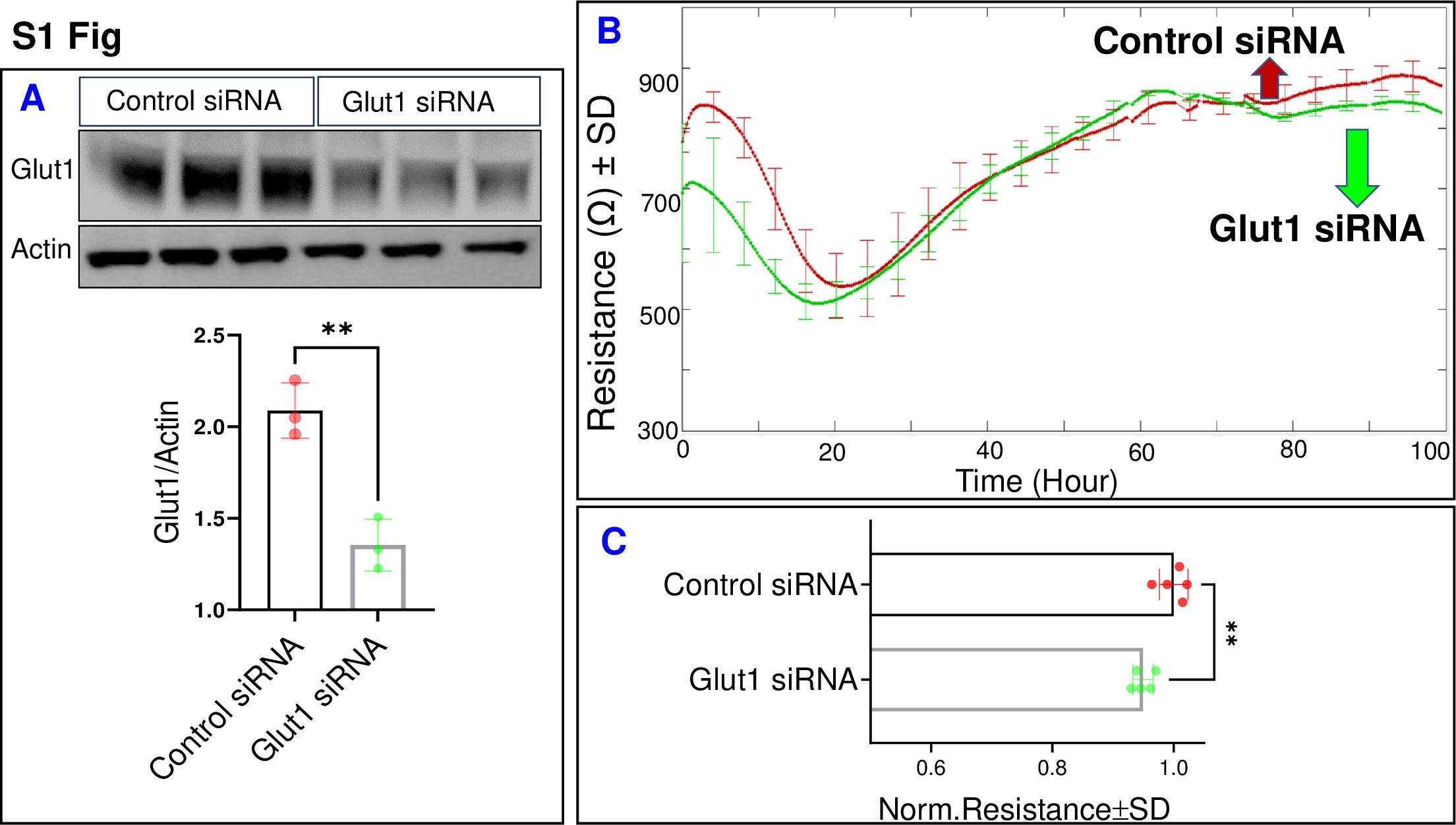

Supplement: S1 Fig — (A) Relative Glut1 expression to actin post-transfection with Glut1 siRNA analyzed by Western blot. (B) The plot illustrates the resistance across HREC monolayers over time, measured at an AC frequency of 4000 Hz, for both the control group transfected with scramble siRNA (10 nM) and HRECs transfected with Glut1 siRNA (10 nM). (C) The bar graph represents normalized resistance for the control group transfected with scramble siRNA (10 nM) and HRECs transfected with Glut1 siRNA (10 nM) at the experiment’s endpoint. Significant changes are denoted by the p-value symbol **<0.01; n = 5 biological replicates for each group. (TIF) [file pone.0294909.s001.tif]

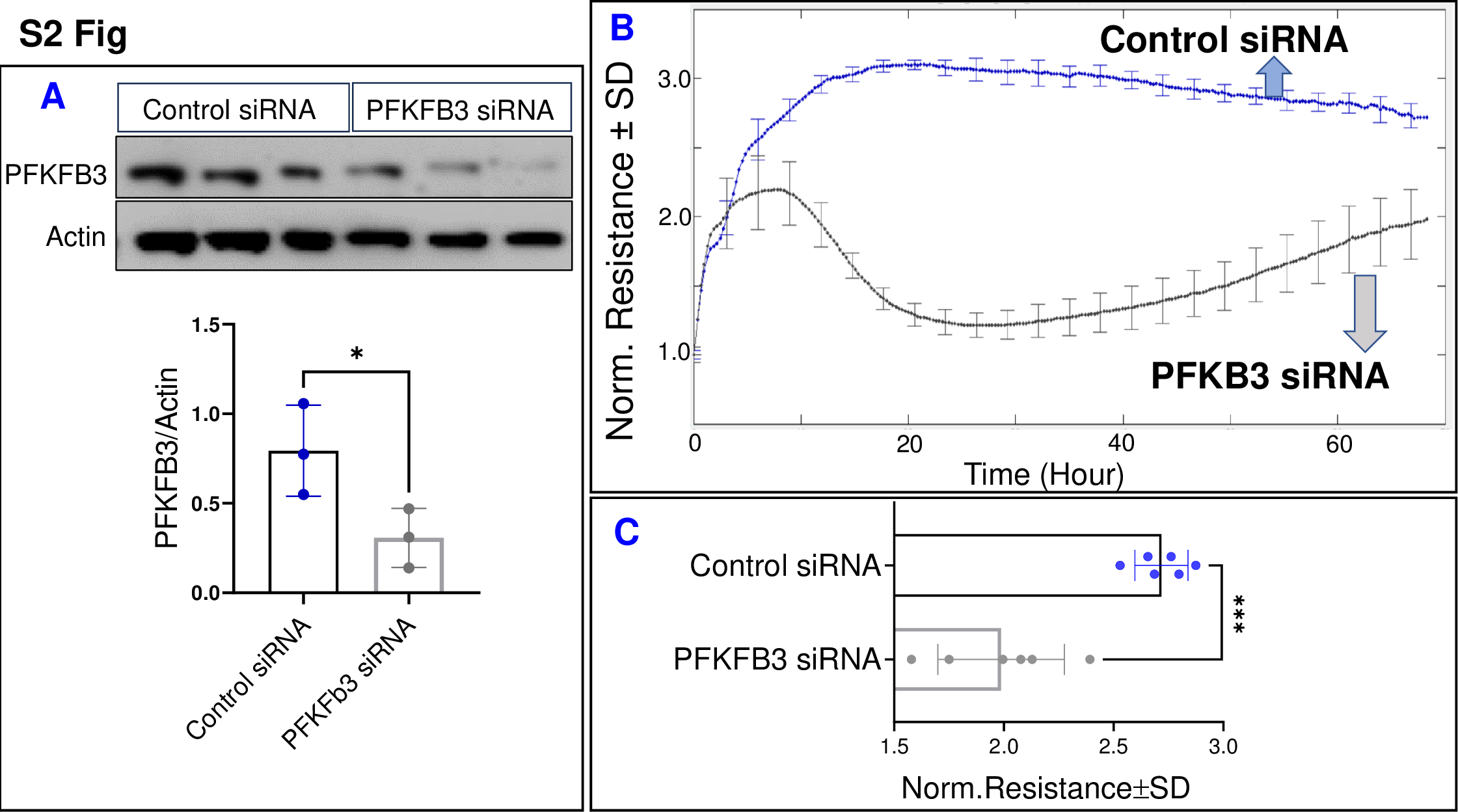

Supplement: S2 Fig — (A) Relative PFKFB3 expression to actin post-transfection with PFKFB3 siRNA analyzed by Western blot. (B) The plot illustrates the resistance across HREC monolayers over time for both the control group transfected with scramble siRNA (10 nM) and HRECs transfected with PFKFB3 siRNA (10 nM). (C) The bar graph represents normalized resistance for the control group transfected with scramble siRNA (10 nM) and HRECs transfected with PFKFB3 siRNA (10 nM) at the experiment’s endpoint. Significant changes are denoted by the p-value symbols *<0.05 and ***<0.001; n = 5 biological replicates for each group. (TIF) [file pone.0294909.s002.tif]

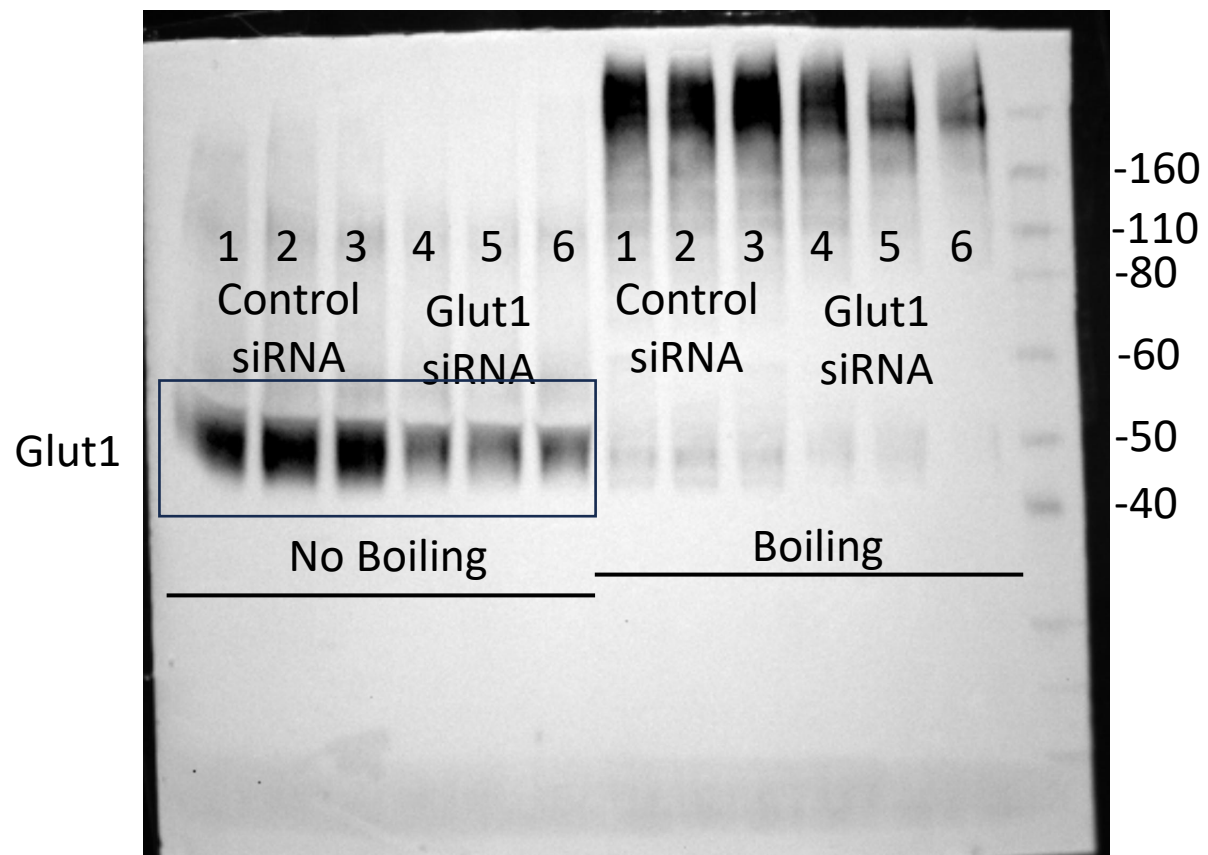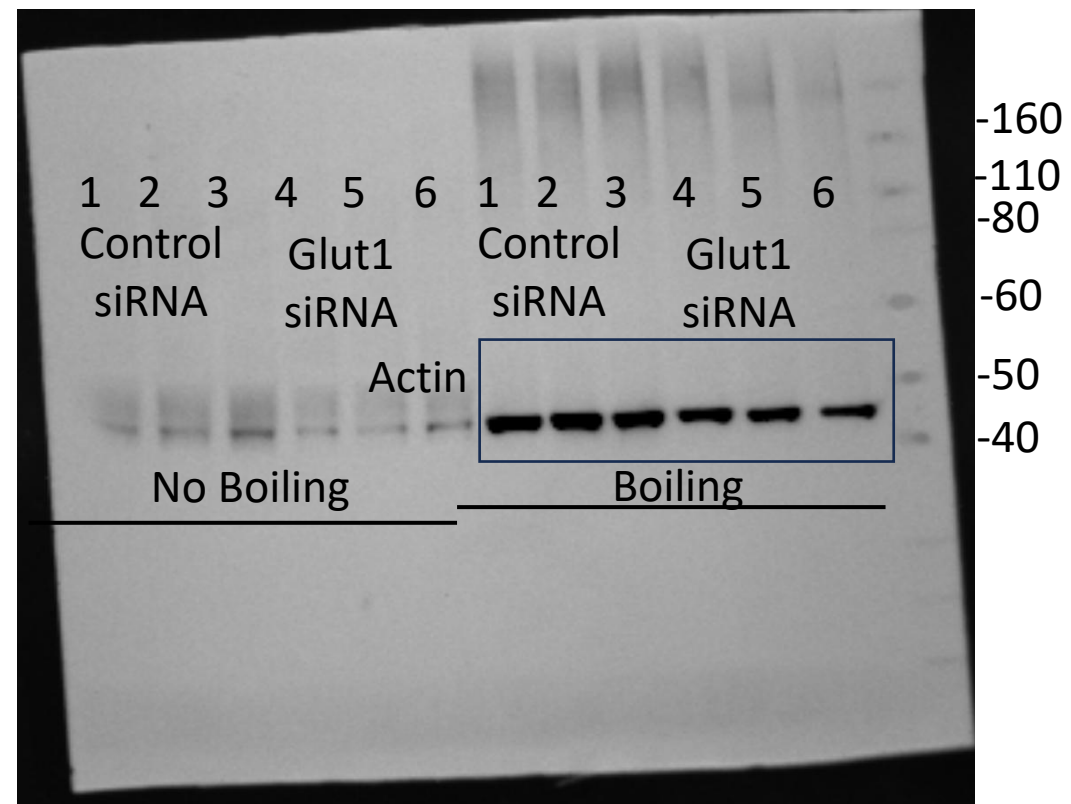

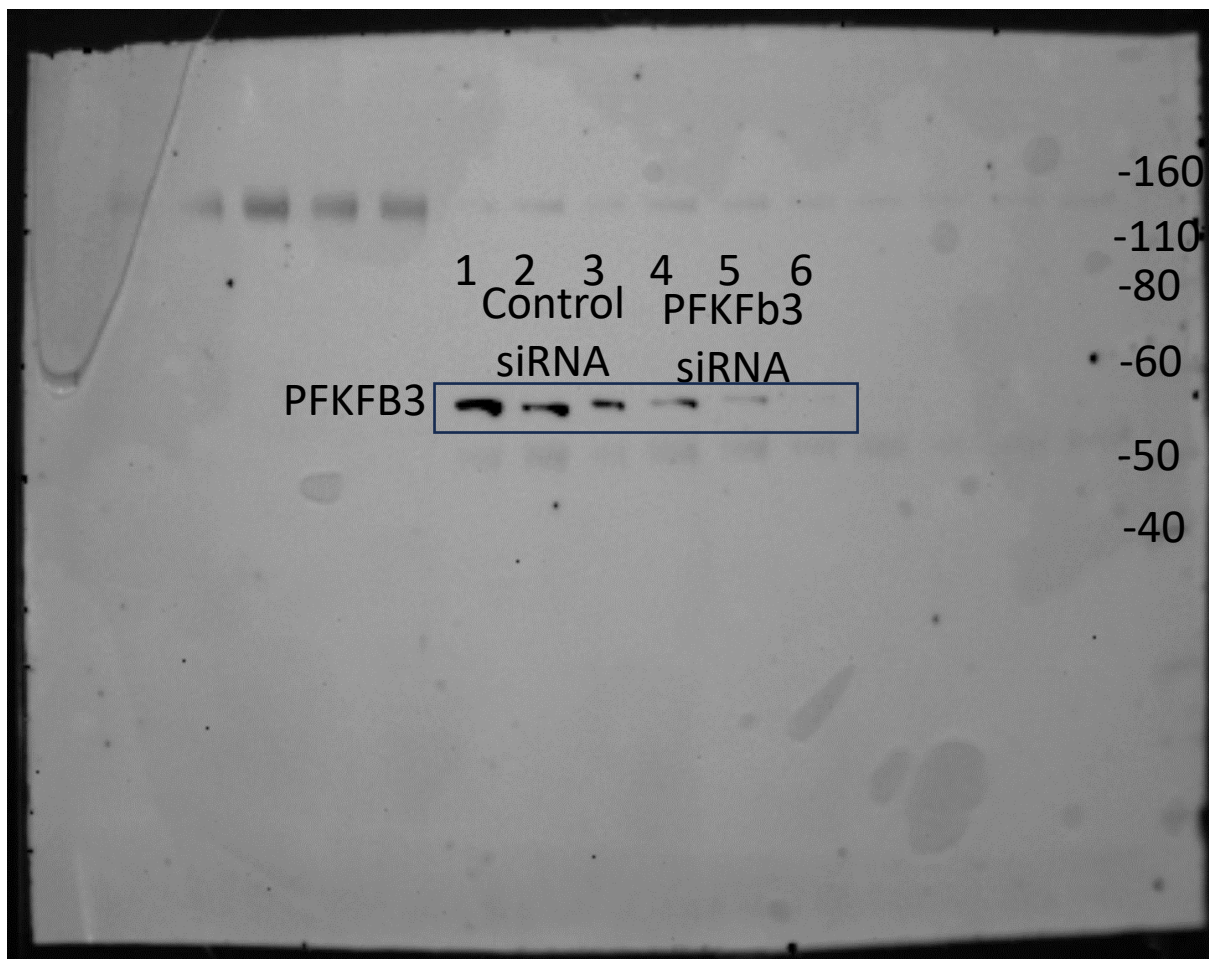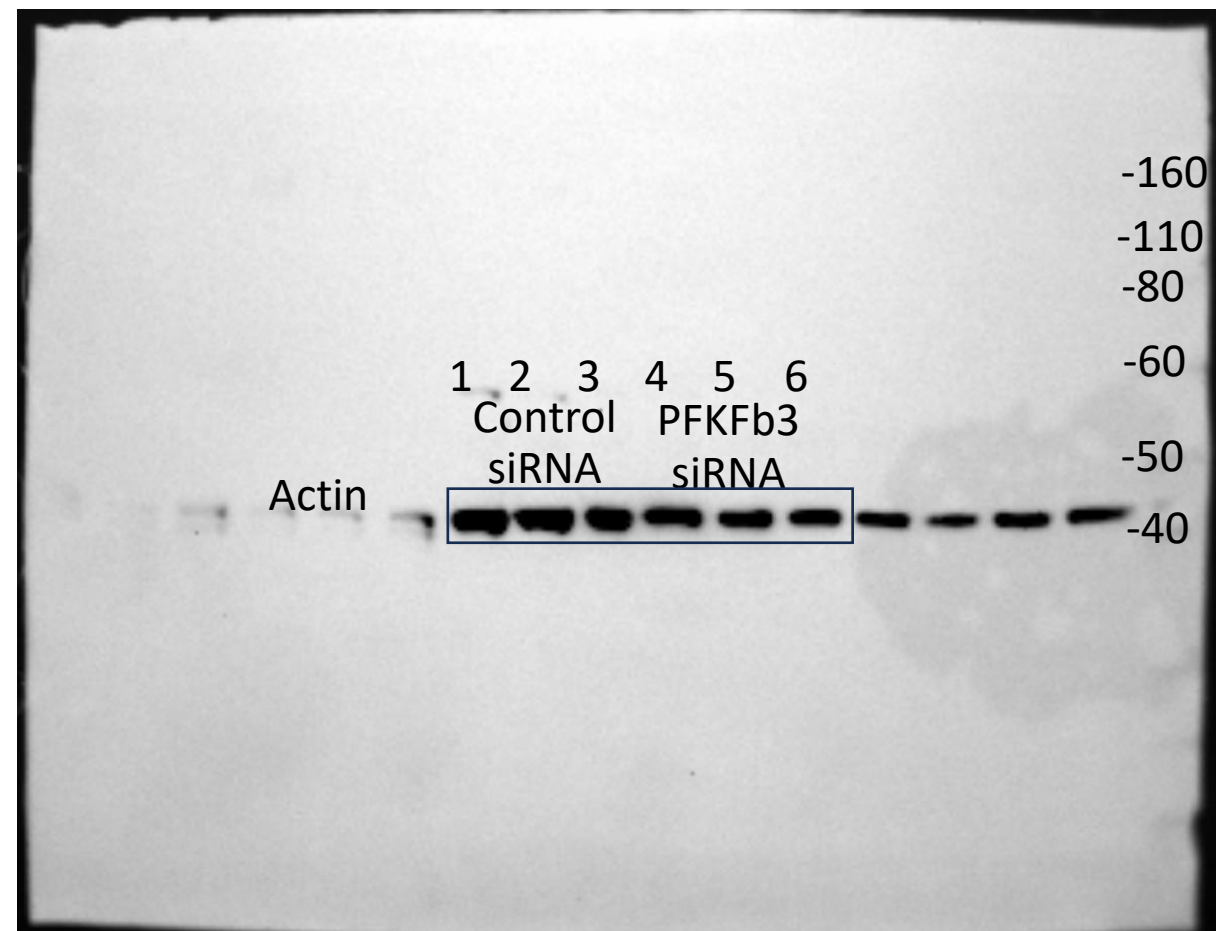

Supplement: S1 Raw images — (PDF) [file pone.0294909.s003.pdf]
